# Trial arm outcome variance difference after dropout as an indicator of missing‐not‐at‐random bias in randomized controlled trials

**DOI:** 10.1002/bimj.202200116

**Published:** 2023-09-20

**Authors:** Audinga‐Dea Hazewinkel, Kate Tilling, Kaitlin H. Wade, Tom Palmer

**Affiliations:** ^1^ Population Health Sciences, Bristol Medical School University of Bristol Bristol UK; ^2^ Medical Research Council Integrative Epidemiology Unit, Bristol Medical School University of Bristol Bristol UK

**Keywords:** bias, dropout, missing‐not‐at‐random, randomized controlled trials, risk‐of‐bias

## Abstract

Randomized controlled trials (RCTs) are vulnerable to bias from missing data. When outcomes are missing not at random (MNAR), estimates from complete case analysis (CCA) and multiple imputation (MI) may be biased. There is no statistical test for distinguishing between outcomes missing at random (MAR) and MNAR. Current strategies rely on comparing dropout proportions and covariate distributions, and using auxiliary information to assess the likelihood of dropout being associated with the outcome. We propose using the observed variance difference across trial arms as a tool for assessing the risk of dropout being MNAR in RCTs with continuous outcomes. In an RCT, at randomization, the distributions of all covariates should be equal in the populations randomized to the intervention and control arms. Under the assumption of homogeneous treatment effects and homoskedastic outcome errors, the variance of the outcome will also be equal in the two populations over the course of follow‐up. We show that under MAR dropout, the observed outcome variances, conditional on the variables included in the model, are equal across trial arms, whereas MNAR dropout may result in unequal variances. Consequently, unequal observed conditional trial arm variances are an indicator of MNAR dropout and possible bias of the estimated treatment effect. Heterogeneous treatment effects or heteroskedastic outcome errors are another potential cause of observing different outcome variances. We show that for longitudinal data, we can isolate the effect of MNAR outcome‐dependent dropout by considering the variance difference at baseline in the same set of patients who are observed at final follow‐up. We illustrate our method in simulation for CCA and MI, and in applications using individual‐level data and summary data.

## 1 INTRODUCTION

Randomized controlled trials (RCTs) are considered the gold standard for assessing the causal effect of an exposure on an outcome, but are vulnerable to bias due to missingness in the outcome—or “dropout.” The impact of dropout depends on the missingness mechanism and the analysis model (Dziura et al., 2013; Little & Rubin, 2020; Rubin, 1976). Three missingness mechanisms can be distinguished: missing completely at random (MCAR), missing at random (MAR), and missing not at random (MNAR) (Rubin, 1976). With MCAR, missingness is unrelated to any measured or unmeasured characteristics and the observed data are a representative subset of the full data. MAR means that the missingness can be explained by observed data and, with MNAR, missingness is a function of the unobserved data.

Two common methods of dealing with dropout are complete case analysis (CCA) and multiple imputation (MI). A CCA is the analysis model intended to be applied to the trial data at its outset, restricted only to individuals with observed outcomes. With MI, missing outcome values are repeatedly imputed conditional on the observed data, generating multiple complete datasets to which the analysis model is applied (Buuren, 2018; Little & Rubin, 2020; Rubin, 1987, 1996), with the resulting estimates subsequently pooled using Rubin's rules (Rubin, 1987). In practice, a CCA will be unbiased if dropout is MCAR or MAR, conditional on the analysis model covariates. MI will be unbiased if dropout is MCAR or MAR conditional on the analysis model and imputation model covariates, and if the imputation model is correctly specified. Generally, both will be biased when outcomes are MNAR (Dziura et al., 2013; Hughes et al., 2019; Little & Rubin, 2020). In this article, we consider the case of an RCT with an incomplete continuous outcome, where we assume that the outcome is generated from covariates and treatment according to a linear model. In such an RCT, a CCA will only be biased if the dropout is related the outcome, conditional on the model covariates (Carpenter & Smuk, 2021; Hughes et al., 2019; White & Carlin, 2010). As RCT analyses typically adjust for a number of baseline covariates, in this article, we primarily focus on (the bias of) the treatment effect, when estimated conditional on some baseline covariate.

In the presence of dropout, observed data generally cannot be used to establish if outcomes are MNAR or MAR given the model covariates. Whether outcomes are MCAR can be partially tested using Little's MCAR test, which compares the multivariate distribution of observed variables of patients with observed outcomes to those with unobserved outcomes (Little, 1988). Little's MCAR test and related tests, however, rely on strong parametric assumptions, with a conclusion that hinges on the specification of some arbitrary *P*‐value cutoff, which limits their practical value (Li & Stuart, 2019). Currently, there is no established statistical test for distinguishing between MAR and MNAR missingness, and consequently, no simple way to determine whether the treatment effect estimate is likely to be biased.

Current guidance for assessing risk of bias due to dropout relies on checking if dropout is differential across trial arms, assessing the plausibility that dropout may be related to outcome (e.g., dropout due to lack of efficacy) (Higgins et al., 2012; Sterne et al., 2019), and comparing the baseline covariate distribution across trial arms in patients who are still observed at the end of follow‐up (Groenwold et al., 2014). While both differential dropout across trial arms and different baseline covariate distributions across trial arms in the observed data can be caused by MNAR dropout, these markers may also result from MAR dropout. The European Medical Agency (EMA) and the National Research Council (NRC), recommend using MAR‐appropriate methods for the primary analysis, followed by sensitivity analyses that weaken this assumption (Clinical Trials o. H. M. D. iNRCUP, 2010; European Medicines Agency (EMA), 2020). These guidelines, however, are in practice implemented in a fraction of all trials, with on average only 6% (of *N* = 330 trials) describing the assumed missing data mechanism (Hussain et al., 2017; Rombach et al., 2015), 9% (*N* = 237) justifying their choice of main analysis (Rombach et al., 2015), and 19% (*N* = 849) reporting some kind of sensitivity analysis (Bell et al., 2014; Hussain et al., 2017; Rombach et al., 2015; Wood et al., 2004; Zhang et al., 2017), which rarely involves relaxing the primary analysis assumptions (Bell et al., 2014), and only 9% (*N* = 200) discussing the risk of bias resulting from missing data (Zhang et al., 2017). This discrepancy between recommended and implemented practice persists despite extensive literature on the subject and may be due to the relative complexity of such analyses.

In this paper, we propose using the differences between the observed variances of the outcome across the two arms of the trial to assess the risk of CCA estimator bias due to MNAR dropout. We show, using directed acyclic graphs (DAGs) and standard statistical theory, how MNAR may give rise to unequal outcome variances between the fully observed subjects in the two arms of the trial. We illustrate this method using individual‐level data and summary‐level data. Individual‐level patient data were obtained from an RCT investigating the benefit of an acupuncture treatment policy for patients with chronic headaches (ISRCTN96537534) (Vickers et al., 2004). The summary data application used published statistics from a cluster‐randomized clinical trial, which investigated psychological outcomes following a nurse‐led preventive psychological intervention for critically ill patients (POPPI, registration ISRCTN53448131).

## 2 NOTATION

Let *U* be some unmeasured covariate, and *C* some measured covariate. Let X=j denote the randomized trial arms, with j={0,1}, and X=0 denoting the comparator arm and X=1 the intervention arm. We define the continuous outcome variable, *Y*, as a linear function of *X*, *U*, and *C* so that

(1) $$Y=\alpha +\beta X+\gamma U+\delta C+\varepsilon_{Y},$$

with α some intercept, β the treatment effect, γ and δ the effects of *U* and *C* on *Y*, respectively, and $\varepsilon_{Y}$ the mean‐zero error term, with $\varepsilon_{Y}$ independent of *X*, *U*, and *C*, and with *U* and *C* additionally independent of *X*.

Let μ_1_ denote the mean of the outcome, *Y*, when X=1, μ_0_ the mean of *Y* when X=0, with

(2) $$\mu_{1}=E\left[Y|X=1\right]=\alpha +\beta +\gamma E\left[U|X=1\right]+\delta E\left[C|X=1\right]+E\left[\varepsilon_{Y}|X=1\right],$$

and

(3) $$\mu_{0}=E\left[Y|X=0\right]=\alpha +\gamma E\left[U|X=0\right]+\delta E\left[C|X=0\right]+E\left[\varepsilon_{Y}|X=0\right].$$

As *C*, *U*, and $\varepsilon_{Y}$ are independent of *X* so that, for example, E[U|X=1]=E[U|X=0], the mean difference across trial arms ($\mu_{1}-\mu_{0}$) reduces to β and we can write:

$$\beta =\mu_{1}-\mu_{0}=E\left[Y|X=1\right]-E\left[Y|X=0\right].$$

We use “full data” to refer to all data that would have been observed on all trial participants, had there been no dropout. “Observed data” refer to all data for the study participants who did not drop out. We define a response indicator *R*, with R=1 when *Y* is observed, and R=0 when *Y* is missing. Let $Y^{*}$ denote the outcome in the observed data and $\beta^{*}$ the treatment effect estimate in the observed data:

(4) $$\beta^{*}=E\left[Y|X=1,R=1\right]-E\left[Y|X=0,R=1\right].$$

The bias, *B*, of the CCA treatment effect estimate is given by the difference of the population treatment effect in the full data (β) and in the observed data ($\beta^{*}$):

(5) $$B=\beta^{*}-\beta .$$

Optionally, the treatment effect in the observed data may be defined conditional on some observed covariate(s), *C*, so that

(6) $$\beta_{C}^{*}=E\left[Y|X=1,C,R=1\right]-E\left[Y|X=0,C,R=1\right],$$

and the bias, $B_{C}$, is given by

(7) $$B_{C}=\beta_{C}^{*}-\beta ,$$

with $\beta_{C}^{*}$ estimated in an ordinary least squares (OLS) regression of the observed outcome, $Y^{\;*}$, on *X* and *C*.

By definition, for the linear model in (1), the population variance of *Y*, for a given trial arm, *j*, in the full data, is given by

(8) $$\begin{array}{cc} \text{var}(Y|X=j)= & \gamma^{2}\text{var}\left(U|X=j\right)+\delta^{2}\text{var}\left(C|X=j\right)+\text{var}\left(\varepsilon_{Y}|X=j\right)+\\ & 2\gamma \;\text{cov}\left(U,\varepsilon_{Y}|X=j\right)+2\delta \;\text{cov}\left(C,\varepsilon_{Y}|X=j\right)+2\gamma \delta \;\text{cov}\left(U,C|X=j\right). \end{array}$$

With *U* and *C* independent of $\varepsilon_{Y}$, all covariance terms involving $\varepsilon_{Y}$ are 0, and (8) reduces to

(9) $$\begin{array}{cc} \text{var}(Y|X=j)= & \gamma^{2}\text{var}\left(U|X=j\right)+\delta^{2}\text{var}\left(C|X=j\right)+\\ & \text{var}\left(\varepsilon_{Y}|X=j\right)+2\gamma \delta \;\text{cov}\left(U,C|X=j\right). \end{array}$$

Let VD denote the outcome variance difference across trial arms in the full data:

(10) VD=var(Y|X=1)−var(Y|X=0).

With *Y* generated according to (1), *U*, *C*, and $\varepsilon_{Y}$ are independent of *X* so that, for example, var(U|X=1)=var(U|X=0), resulting in an expected outcome variance difference of 0 in the full data. The assumptions necessary for this to hold are discussed further in Section 3.

In the observed data, the outcome variance in a given trial arm, *j*, is given by

$$\begin{array}{cc} \text{var}(Y|X=j,R=1)= & \gamma^{2}\text{var}\left(U|X=j,R=1\right)+\delta^{2}\text{var}\left(C|X=j,R=1\right)+\\ & \text{var}\left(\varepsilon_{Y}|X=j,R=1\right)+2\gamma \;\text{cov}\left(U,\varepsilon_{Y}|X=j,R=1\right)+\\ & 2\delta \;\text{cov}\left(C,\varepsilon_{Y}|X=j,R=1\right)+2\gamma \delta \;\text{cov}\left(U,C|X=j,R=1\right), \end{array}$$

and the variance difference across trial arms by

(11) $$\begin{array}{c} {\text{VD}}^{*}=\text{var}\left(Y|X=1,R=1\right)-\text{var}\left(Y|X=1,R=1\right). \end{array}$$

The exact form of (11) is determined by the relationship between the covariates, outcome, and *R*, and is explored in Section 3. Again, the variance may be defined conditional on *C*, with the variance difference in the full data then given by

(12) $${\text{VD}}_{C}=\text{var}\left(Y|X=1,C\right)-\text{var}\left(Y|X=0,C\right),$$

and, in the observed data, by

(13) $${\text{VD}}_{C}^{*}=\text{var}\left(Y|X=1,C,R=1\right)-\text{var}\left(Y|X=0,C,R=1\right).$$

We can estimate (13) using the studentized Breusch–Pagan estimator, detailed further in Section 4. In the next section (Section 3), we show under which dropout mechanisms the variance difference across trial arms in the observed data can be expected to be different from 0.

## 3 MAR AND MNAR DROPOUT AND OUTCOME VARIANCES ACROSS TRIAL ARMS IN THE OBSERVED DATA

In an RCT, patients are randomized to treatment, after which treatment is initiated and the patients are followed up over a period of time, during which dropout may occur. Randomization makes it plausible to assume that the trial arms have equal outcome variances prior to treatment initiation in the full data. Given two additional assumptions, we can expect this to hold after treatment initiation and throughout follow‐up:

Assumption A1 There is no treatment effect heterogeneity so that the treatment effect, β, is the same for every individual.

Assumption A2 The errors are homoskedastic so that the error term of the outcome (1), $\varepsilon_{Y}$, does not depend on treatment or on *Y* itself.

If these two assumptions hold, then the trial arm population outcome variances can be expected to remain the same throughout follow‐up in the full data. Then, it follows that if dropout is present and the trial arm outcome variances are different in the observed data, this must be due to dropout. Here, we use directed acyclic graphs (DAGs) to describe different MAR and MNAR dropout mechanisms, and show, using graphical model theory (Mohan & Pearl, 2021), that the trial arm variances, conditional on the model covariates, are the same when dropout is MAR, but may be different when dropout is MNAR. Additionally, we show, for an outcome, *Y* (Equation (1)), generated according to a linear model, that certain types of MNAR dropout do not result in a biased treatment effect estimate or different trial arm outcome variances. We define bias, $B_{C}$ (7) with respect to a treatment effect, $\beta_{C}^{*}$ (6), estimated while adjusting for some observed baseline covariate, *C*, which is a predictor of *Y* (Equation (1)). Analogously, we define the variance difference as the difference in trial arm outcome variances, conditional on *C*, denoted as VD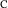 (12) and VD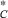 (13) in the full and observed data, respectively. Let P(Y|X,C) denote the density of *Y*, conditional on *X* and some observed baseline covariate, *C*, in the full data, and P(Y|X,C,R=1) the corresponding density in the observed data.

Proposition 1 The densities P(Y|X,C) and P(Y|X,C,R) will be identical only when dropout is MCAR or MAR, with *R* independent of *Y* given the variables included in the analysis model (R⊥⊥Y|X,C). Any quantities derived from the densities, such as the mean difference and variance difference across *X*, will be also the same. If assumptions A1 and A2 are satisfied so that the variances of the outcome in the two trial arms are equal in the full data, then P(Y|X,C)=P(Y|X,C,R) implies that the variances of the outcome in the two trial arms are also equal in the observed data.

**FIGURE 1:**
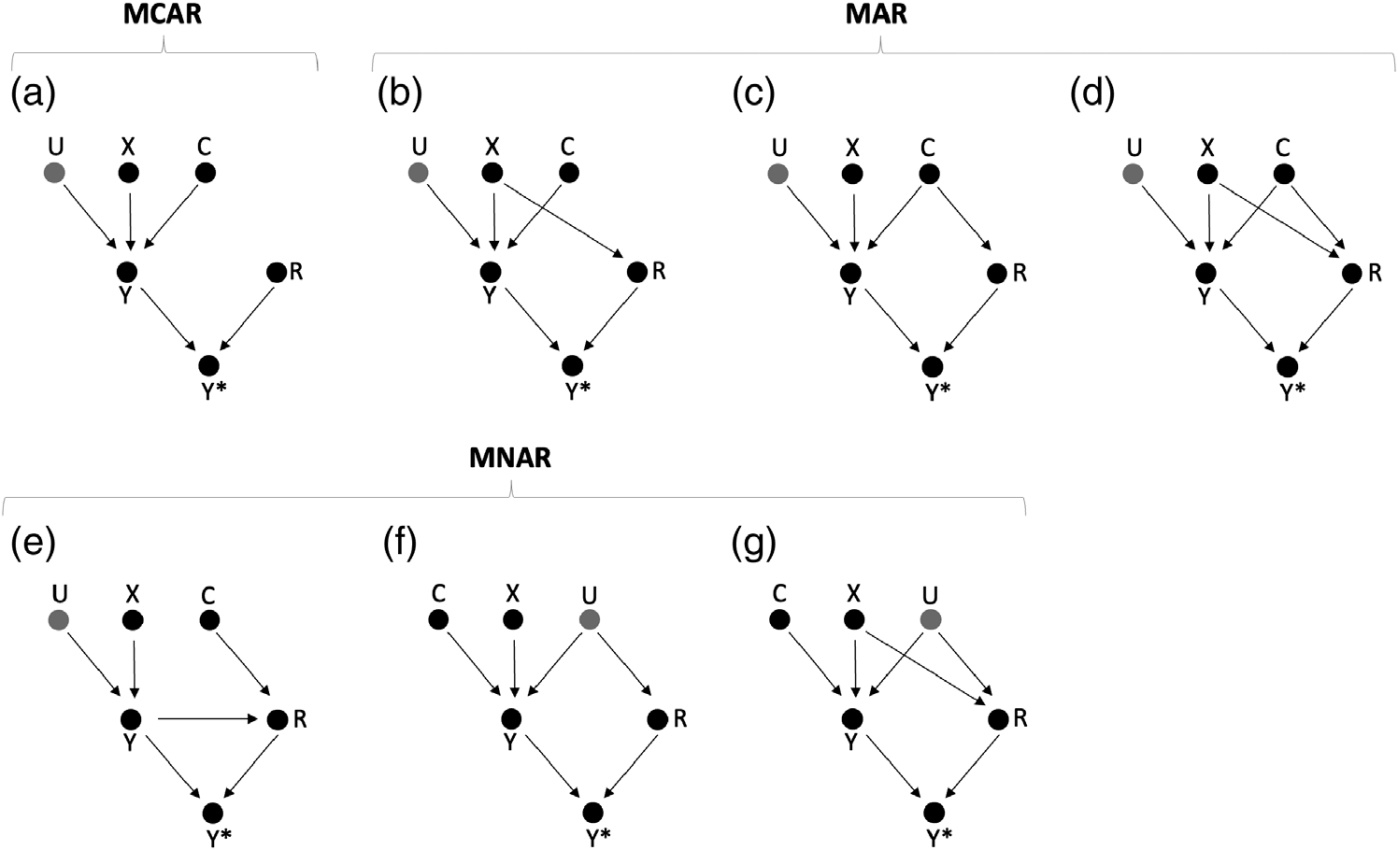
Seven directed acyclic graphs (DAGs), depicting the relationship between binary treatment (*X*), continuous outcome (*Y*), an unmeasured continuous variable (*U*), a measured continuous variable (*C*), and the response indicator (*R*). The observed outcome is some function f(Y,R) and denoted $Y^{*}$. The dropout mechanisms—missing completely at random (MCAR), missing at random (MAR) and missing not at random (MNAR)—are defined with respect to an analysis model that regresses the observed outcome, $Y^{*}$, on *X* and *C*.

In Figure 1(a), dropout is MCAR, with the response indicator, *R*, unaffected by any observed or unobserved variables. Figures 1(b)–(d) depict MAR dropout mechanisms, with dropout dependent on treatment, *X*, on some baseline covariate, *C*, and on both *X* and *C*, respectively. In all four scenarios, *R* is independent of *Y* given *C* and *X*, and we can show that the density of *Y*, conditional on *X* and *C*, in the full data, is equal to the corresponding density of *Y* in the observed data: P(Y|X,C)=P(Y|X,C,R=1) (proofs given in Online Appendix A.1). This has the following implications. First, the outcome mean difference across trial arms conditional on *C*, $\beta_{C}^{*}$ (6), will be the same in the full and observed data so that the CCA treatment effect estimate is unbiased, with $B_{C}=0$. Second, if the outcome variances are equal in X=1 and X=0 in the full data, then the outcome variances in the observed data can also be expected to be equal across trial arms. The latter requires that assumptions A1 and A2 are satisfied so that that the treatment effects are homogeneous and the outcome errors homoskedastic.

Figures 1(e)–(g) depict MNAR dropout mechanisms, with dropout dependent on outcome, *Y*, on some unobserved covariate, *U*, and on treatment, *X*, and *U* both. Then, for all three scenarios, we can show that *R* is not independent of *Y* given the covariates included in the analysis model, and consequently, that P(Y|X,C)≠P(Y|X,C,R=1) (proofs given in Online Appendix A.1). Different densities in the observed and full data imply that the outcome means and variances can be different also so that the CCA treatment effect estimate is biased and the outcome variance difference across trial arms nonzero. However, as we assume that *Y* (Equation (1)) is generated according to a linear model, an exception to this rule arises for MNAR dropout that occurs according to scenario of Figure 1(f).

Proposition 2 Let the outcome, *Y*, be defined as in (1). If dropout depends on unmeasured covariate, *U*, and if (U⊥⊥X)|R, then the outcome mean difference and variance difference across trial arms in the full data can be estimated from the observed data, without conditioning on *U*. If assumptions A1 and A2 are satisfied so that the variances of the outcome in the two trial arms are equal in the full data, this implies that the variances of the outcome in the two trial arms are also equal in the observed data.

For all scenarios in Figure 1, we assume that the measured and unmeasured covariates are independent of treatment (C⊥⊥X and U⊥⊥X), which can be expected to hold in a randomized trial setting. Under the additional assumption of homoskedastic errors (assumption A2), $\varepsilon_{Y}⊥\;\;\;⊥X$. Then, the treatment effect estimate, β, can be estimated by the unconditional mean difference across trial arms, as in (2). If we also assume that the treatment effects are homogeneous (A1), all the variance components in (9) can be expected to be equal across trial arms (e.g., var(U|X=0)=var(U|X=1)) so that outcome variance difference across trial arms in the full data, VD (10), is 0. If these independencies also hold in the observed data (U⊥⊥X|R, C⊥⊥X|R, and $\varepsilon_{Y}⊥\;\;\;⊥X|R$), then the unconditional treatment effect estimate in the observed data, $\beta^{*}$ (4), is equal to β (2) so that the bias of the CCA treatment effect estimate, B=0 (5), and the outcome variance difference across trial arms, in the observed data, VD* (11), is also 0.

In Figure 1(f), dropout depends on unmeasured covariate, *U*. While this is an MNAR dropout mechanism, it results in an unbiased CCA treatment effect estimate and no outcome variance difference across trial arms, as *U* is independent of *X* in the observed data: U⊥⊥X|R (proof given in Online Appendix A.2). While the same reasoning could be applied to Figure 1(g), where dropout is MNAR dependent on *X* and *U*, this, however, would require the additional assumption that the effects of *X* and *U* on *R* are independent (e.g., sicker people drop out but equally so in both trial arms). For the purposes of this paper, we do not make this assumption, and allow *X* and *U* to interact (e.g., sicker people drop out but more so in the comparator arm). Then, in the observed data, *X* and *U* are no longer independent (X¬⊥⊥U|R). Consequently, $\beta^{*}$ will be biased (B≠0), and VD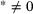. Formulae for $\beta^{*}$, *B*, and VD* are given in Online Appendix A.2 (Equations (A.13), (A.14), and (A.16), respectively). Formulae for the bias and outcome variance difference when conditioning on *C*, $\beta_{C}^{*}$ (6) and VD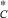 (13) are given in Equations (A.15) and (A.17). Note that if *U* and *C* are related, this will additionally mean that also X¬⊥⊥C|R. Conditioning on *C* will result in attenuated estimates of the CCA estimator bias, $B_{C}$, and the outcome variance difference across trial arms in the observed data, VD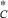, when compared to *B* and VD*, respectively. When *C* and *U* are independent, however, conditioning on *C* will leave both estimates unaffected.

In summary, when dropout is dependent only on some covariate that is either unobserved or excluded from the model, this will not, for a linear model of *Y*, result in a biased CCA treatment effect estimate or in an outcome variance difference across trial arms in the observed data, even though such dropout is strictly speaking MNAR. When dropout depends on both some unmeasured covariate and *X*, and they are not independent in the observed data, this may result in bias and a variance difference. Table 1 provides an overview of when the seven dropout scenarios of Figure 1 result in a biased CCA estimate and an outcome variance difference across trial arms in the observed data, for a linear regression of *Y* on *X* and *C*.

**TABLE 1:** Dropout mechanism, type according to Rubin (1976), bias of the complete case analysis (CCA) estimate, and variance difference (VD) across trial arms in the observed data for the seven directed acyclic graphs (DAGs) of Figure 1. We assume an outcome, *Y*, generated according to a linear model as in (1). The characteristics are defined with respect to models that adjust for a measured baseline covariate, *C*. “Bias” refers to the CCA estimator bias for an analysis adjusted for *C*. VD refers to the outcome variance difference across trial arms in the observed data, estimated conditional on *C*. “Type” is defined with respect to the covariates included in the analysis model; *X* and *C*.

Scenario	Dropout mechanism	Type	Bias	VD 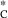
A	Random	MCAR	No	No
B	*X*‐dependent	MAR	No	No
C	*C*‐dependent	MAR	No	No
D	*X*‐ and *C*‐dependent	MAR	No	No
E	*Y*‐dependent	MNAR	Yes	Yes
F	*U*‐dependent	MNAR	No	No
G	*X*‐ and *U*‐dependent	MNAR	Yes	Yes

MAR, missing at random; MCAR, missing completely at random; MNAR, missing not at random.

Note that the seven dropout scenarios of Figure 1 and Table 1 are illustrative settings and that we do not provide a comprehensive review of all possible settings. For example, dropout may simultaneously depend on treatment, *X* (scenario B), some observed covariate, *C*, (scenario C), and on the outcome, *Y* (scenario E). If part of the dropout mechanism depends on *Y* (scenario E) or both *X* and *U* (scenario G), this is generally sufficient to cause a biased CCA estimate and an outcome variance difference across trial arms in the observed data. Under assumptions A1 and A2, an outcome variance difference across trial arms serves as a marker of MNAR dropout that may result in bias. However, such MNAR dropout will not always result in an outcome variance difference, which may, because of several biases acting in different directions, be very small or 0. For example, dropout may depend on the outcome in such a way that the top quartile of the intervention arm and bottom quartile of the control arm drop out. Such a setting would result in a biased CCA estimate but no outcome variance difference across trial arms.

## 4 METHODS FOR TESTING DIFFERENCES IN VARIANCES

Various methods are available for testing and estimating the difference in variance between two groups, including Bartlett's test (Bartlett & Fowler, 1937), Levene's test (Levene at al., 1960), the Brown–Forsythe test (Brown & Forsythe, 1974), the Breusch–Pagan test (Breusch & Pagan, 1979), and the studentized Breusch–Pagan test (Koenker, 1981). In this paper, we employ the latter, as it has a straightforward implementation that allows for conditioning on additional covariates, and is also robust against nonnormally distributed errors. This is particularly relevant, as, in practice, outcomes are unlikely to be strictly normally distributed.

The studentized Breusch–Pagan estimate is obtained as follows. First, the outcome, *Y*, is regressed on the treatment variable, *X*, and optional additional covariates, *C*, in an OLS regression:

$$Y=\alpha +\hat{\beta }X+\gamma C+\varepsilon_{Y}.$$

The regression residuals are obtained and squared (${\hat{\varepsilon }}_{Y}^{2}$) and, in a second auxiliary OLS regression, regressed on the treatment variable:

$${\hat{\varepsilon }}_{Y}^{2}=\delta +\kappa X+\eta_{\varepsilon_{Y}}.$$

The variance difference estimate is given by $\hat{\kappa }$, and the test statistic is given by $nR^{2}$, with *n* the sample size and *R* ^2^ the coefficient of determination, obtained from the second auxiliary regression.

## 5 MNAR DROPOUT AND HETEROGENEOUS TREATMENT EFFECTS IN LONGITUDINAL DATA

In Section 3, we showed that outcome‐dependent MNAR dropout and MNAR dropout dependent on *X* and *U*, with *U* some unmeasured predictor of *Y*, can result in a biased treatment effect estimate and an outcome variance difference across trial arms in the observed data, whereas MAR dropout will result in neither, subject to assumptions A1 and A2. When A1 and A2 hold so that the treatment effects are homogeneous and the outcome errors are homoskedastic, the expected variance difference across trial arms in the full data is 0, which implies that a variance difference across trial arms in the observed data can be used as a marker of MNAR dropout and bias. Treatment effect heterogeneity and heteroskedastic outcome errors, however, will result in a nonzero variance difference across trial arms in both the full and observed data so that MNAR dropout is no longer the only potential cause of a variance difference. Treatment effect heterogeneity can be investigated by checking for the presence of an effect modifier, for example, by performing a stratified analysis. Heteroskedastic outcome errors can be investigated by exploring if the variability in the outcome at baseline is different for patients with lower and higher values. We elaborate on this in the applied example of Section 7.1.

Here, we examine the implications of violating assumption A1 through the introduction of effect modification, which will result in a nonzero expected outcome variance difference across trial arms in the full data and in the observed data (i.e., patients with outcomes at follow‐up observed). We show that when this assumption is violated, for longitudinal data, where the outcome is measured in a time series, the presence of MNAR dropout can still be assessed by looking at the outcome variance difference across trial arms in the outcome measured at the baseline.

### 5.1 Outcome variances across trial arms when assumption A1 is violated

Let $Y_{b}$ denote the outcome measured at baseline, prior to treatment initiation, which is a function of covariates *U* and *C*:

(14) $$Y_{b}=\alpha_{b}+\gamma_{b}U+\delta_{b}C+\varepsilon_{b},$$

with $\varepsilon_{b}$ the error term. $Y_{b}$ is unaffected by *X*, and with *U* and *C* independent of $\varepsilon_{b}$, the baseline outcome variance for a given trial arm, *j*, is

(15) $$\begin{array}{cc} \text{var}(Y_{b}|X=j)= & \gamma_{b}^{2}\text{var}\left(U|X=j\right)+\delta_{b}^{2}\text{var}\left(C|X=j\right)+\\ & \text{var}\left(\varepsilon_{b}|X=j\right)+2\gamma_{b}\delta_{b}\;\text{cov}\left(U,C|X=j\right), \end{array}$$

and the outcome variance difference across trial arms at baseline is 0:

(16) $${\text{VD}}_{b}=\text{var}\left(Y_{b}|X=1\right)-\text{var}\left(Y_{b}|X=0\right)=0.$$

Let $Y_{f}$ denote the outcome at follow‐up, which is a function of the baseline outcome, $Y_{b}$, intervention, *X*, covariates *U* and *C*, and effect modifier, *S*:

(17) $$Y_{f}=Y_{b}+\beta X+\gamma U+\delta C+\zeta SX+\varepsilon_{f},$$

with $\varepsilon_{f}$ the error term, which is correlated with $\varepsilon_{b}$. In (17), *S* modifies the effect of *X* on $Y_{f}$, with ζ the effect of *S* on the outcome at follow‐up, $Y_{f}$, in the intervention arm, and the average treatment effect, $\beta_{av}$, given by

(18) $$\beta_{av}=\beta +\zeta .$$

For simplicity, in (17), we do not specify a main effect of *S*, and assume that the effect modification is limited to the intervention arm. More generally, an effect modifier can be expected to modify the outcome in both treatment arms. As for (1), we here assume that *X*, *U*, and *C* are independent of $\varepsilon_{f}$, and that *U* and *C* are independent of *X*, ,but allow *U* and *C* to be dependent. We make the same assumption for *S*, but now assume, for simplicity, that *S* is independent of *U* and *C*. The population variance of $Y_{f}$ in the full data, for the comparator arm, is then given by

(19) $$\begin{array}{cc} \text{var}(Y_{f}|X=0)= & \;{\left(\gamma_{b}+\gamma \right)}^{2}\text{var}\left(U|X=0\right)+{\left(\delta_{b}+\delta \right)}^{2}\text{var}\left(C|X=0\right)\;+\\ & \text{var}\left(\varepsilon_{b}|X=0\right)+\text{var}\left(\varepsilon_{f}|X=0\right)+2\text{cov}\left(\varepsilon_{b},\varepsilon_{f}|X=0\right)+\\ & 2\left(\gamma +\gamma_{b}\right)\left(\delta +\delta_{b}\right)\;\text{cov}\left(U,C|X=0\right), \end{array}$$

and, for the intervention arm, by

(20) $$\begin{array}{cc} \text{var}(Y_{f}|X=1)= & \;{\left(\gamma_{b}+\gamma \right)}^{2}\text{var}\left(U|X=1\right)+{\left(\delta_{b}+\delta \right)}^{2}\text{var}\left(C|X=1\right)\;+\\ & \text{var}\left(\varepsilon_{b}|X=1\right)+\zeta^{2}\text{var}\left(S|X=1\right)+\text{var}\left(\varepsilon_{f}|X=1\right)+\\ & 2\text{cov}\left(\varepsilon_{b},\varepsilon_{f}|X=1\right)+2\left(\gamma +\gamma_{b}\right)\left(\delta +\delta_{b}\right)\;\text{cov}\left(U,C|X=1\right). \end{array}$$

With *U*, *C*, $\varepsilon_{b}$, and $\varepsilon_{f}$ independent of *X*, (19) and (20) result in a variance difference in the outcome at follow‐up across trial arms in the full data of

(21) $$\begin{array}{cc} {\text{VD}}_{f}= & \text{var}\left(Y_{f}|X=1\right)-\text{var}\left(Y_{f}|X=0\right)=\zeta^{2}\text{var}\left(S|X=1\right). \end{array}$$

When ζ≠0 in (17) so that *S* acts as an effect modifier, assumption A1 is violated and the outcome variance difference across trial arms at follow‐up (21) is nonzero. As S⊥⊥C, the covariate‐adjusted variance difference, VD
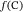
=VD_*f*_. The derivations of (19)–(21) are given in full in Online Appendix B.1. Note that if we allow for a dependency between *S*, *U*, and *C*, (20) and (21) will include additional covariance terms, with, for example, for *S* and *U*, the term $2\zeta \left(\gamma +\gamma_{b}\right)\;\text{cov}\left(S,U|X=1\right)$ (see Online Appendix B.1, Equations (B.7) and (B.8)). Also note that while we omit a main effect of *S* in (17), including it will not affect the expected bias or variance difference, as S⊥⊥X.

In the presence of heterogeneous treatment effects, the outcome variance difference across trial arms in the full data, VD_*f*_ (21), is nonzero. Then, the outcome variance difference across trial arms in the observed data, VD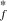, will also be nonzero, irrespective of the dropout mechanism, and consequently, cannot be used to assess the risk of MNAR dropout. In contrast, treatment effect heterogeneity will not result in an outcome variance difference at baseline, in either the full data (VD_*b*_, 16) or the observed data (VD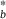), as $Y_{b}$ is not affected by treatment. Outcome‐dependent dropout, however, may result in an outcome variance difference at baseline in the observed data, as the outcome errors at baseline, $\varepsilon_{b}$, and at follow‐up, $\varepsilon_{f}$, are correlated. Consequently, unlike VD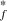, VD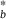 can be used to assess the risk of MNAR dropout and, by extension, CCA estimator bias, when assumption A1 is violated. Similarly, if assumption A2 does not hold, for example, when the error term depends on the outcome, this will, given that a treatment effect is present, result in a variance difference across trial arms in the full data only in the outcome at follow‐up and not at baseline (derivations are given in Online Appendix B.2). In the simulation below, we explore the implications of violating assumption A1 and show that, for longitudinal data, the outcome variance difference across trial arms at baseline, in the observed data, can be used to distinguish between treatment effect heterogeneity and outcome‐dependent dropout.

### 5.2 Methods

We performed a simulation study with the outcome at follow‐up, $Y_{f}$, simulated according to (17), the outcome at baseline, $Y_{b}$, simulated according to (14), and with 1000 patients randomly assigned to each trial arm. The errors of $Y_{b}$ and $Y_{f}$, $\varepsilon_{b}$ and $\varepsilon_{f}$, were drawn from a multivariate normal distribution, with variances of 1.5 and 2, respectively, and a correlation coefficient of 0.433. Dropout was simulated according to the seven mechanisms listed in Table 1, 2 and illustrated in Figure 1, under a logit model, with 28% overall dropout. Each scenario was simulated without effect modification (ζ=0), with a true treatment effect β=1 in the full data, and also with effect modification (ζ=0.5), with the average treatment effect in the full data, $\beta_{av}$ (18), also 1.

The outcome variance difference across trial arms in the observed data was calculated at final follow‐up (VD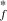) and, in the same set of patients (i.e., patients with observed outcomes at follow‐up), at baseline (VD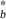). CCA estimator bias and VD estimates were obtained conditional on observed baseline covariate, *C*, (VD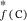) and when additionally adjusting for $Y_{b}$ (VD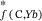). For each scenario, we obtained mean estimates of the CCA estimator bias, VD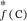, VD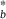, and VD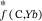, with corresponding 95% confidence intervals (CIs). The 95% CIs were computed using the standard deviation (SD) of the relevant estimate across simulations. We simulated 1000 datasets of N=2000, having verified, for each estimate, that the Monte Carlo SD (MCSD) and mean standard error were comparable, indicating that 1000 repetitions are sufficient. Additionally, we calculated the proportion of times the null was excluded from the confidence interval, as an indicator of how often a variance difference was correctly identified across simulations. A full description of the simulation framework, in accordance with ADEMP guidelines (Morris et al., 2019), is given in Online Appendix C.1, where we describe, in detail, the simulation aims, data‐generating mechanisms, methods, and performance measures.

### 5.3 Results

The simulation results are shown in Table 2. We first consider the seven scenarios when there is no effect modification and the treatment effects are homogeneous (A–G). When dropout is MCAR (A), and when dropout is MAR conditional on treatment (B), some measured covariate (C), or both (D), the treatment effect estimates are unbiased, irrespective of conditioning on $Y_{b}$, with, on average, a zero outcome variance difference across trial arms in the observed data at baseline (VD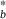) and at follow‐up (VD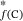). We observe the same for scenario F, where dropout is MNAR dependent on *U*, but with *U* and *X* independent in the observed data. In scenario G, where dropout is MNAR dependent on *U* and *X*, with *U* and *X* not independent in the observed data, the CCA treatment effect estimate is biased, and the mean outcome variance difference across trial arms at follow‐up is nonzero. When dropout is MNAR dependent on $Y_{f}$ (E), we observe a biased treatment effect estimate and nonzero outcome variance differences at both baseline and at follow‐up. Conditioning on $Y_{b}$ results in an attenuated bias estimate and a smaller variance difference at follow‐up (VD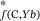). When effect modification is present (EM), resulting in treatment effect heterogeneity, we observe a variance difference at follow‐up, regardless of dropout mechanism. In contrast, we only observe a VD at baseline in scenario E, where dropout is MNAR dependent on outcome. In Online Appendix C.2, a companion table (Table C.1) is provided for Table 2, with additional measures of simulation performance.

**TABLE 2:** Bias of the complete case analysis (CCA) treatment effect estimate, obtained in a linear regression of $Y_{f}$ on *X*, *C*, and, optionally, $Y_{b}$, with 95% confidence intervals (CIs); variance difference (VD) across trial arms in the outcome at baseline, in the observed data (VD); VD across trial arms in the outcome at follow‐up, estimated conditional on *C* (VD), and on *C* and $Y_{b}$ (VD). For each VD estimate, the 95% CI are given, and the proportion of times the null hypothesis of no variance difference across trial arms was rejected (e.g., $p_{b}$, for VD_*b*_). The simulated data are longitudinal, with baseline ($Y_{b}$) and follow‐up ($Y_{f}$) measurements, simulated under seven different dropout mechanisms (see Table 1 and Figure 1), with and without effect modification (EM). The estimates shown are mean values across 1000 simulated datasets of N=2000, with, in the absence of effect modification, a true treatment effect β=1, and trial arm outcome variances at baseline and follow‐up of 2.125 and 10.625, respectively. In the presence of effect modification, $\beta_{av}=1$, and the outcome variance at follow‐up in the intervention arm is 11.625. In bold, we show the point estimates that theory suggests should not be zero.

Unadjusted for outcome at baseline					Adjusted for outcome at baseline			
	Bias (95% CI)	VD 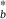 (95% CI)	$p_{b}$	VD 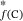 (95% CI)	$p_{\text{(f,C)}}$	Bias (95% CI)	VD 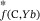 (95% CI)	$p_{\text{f(C,Yb)}}$
*Random dropout*								
A	0.00 (−0.33,0.33)	0.00 (−0.30,0.30)	0.04	−0.01 (−1.37,1.35)	0.04	0.00 (−0.16,0.17)	0.00 (−0.36,0.36)	0.05
A(EM)	0.00 (−0.33,0.34)	0.00 (−0.30,0.30)	0.04	**1.00** (−0.44,2.44)	0.26	0.00 (−0.18,0.18)	**1.00** (0.57,1.44)	1.00
*X* *‐dependent dropout*								
B	0.00 (−0.34,0.34)	0.00 (−0.31,0.30)	0.04	0.01 (−1.38,1.39)	0.03	0.00 (−0.17,0.17)	0.01 (−0.37,0.38)	0.06
B(EM)	0.00 (−0.34,0.34)	0.00 (−0.31,0.30)	0.04	**1.01** (−0.42,2.43)	0.25	0.00 (−0.18,0.17)	**1.01** (0.58,1.43)	1.00
*C* *dependent dropout*								
C	0.00 (−0.32,0.32)	0.00 (−0.31,0.30)	0.04	−0.01 (−1.38,1.35)	0.05	0.00 (−0.15,0.16)	0.00 (−0.35,0.36)	0.05
C(EM)	0.00 (−0.32,0.32)	0.00 (−0.31,0.30)	0.04	**0.99** (−0.42,2.40)	0.27	0.00 (−0.17,0.17)	**1.00** (0.58,1.43)	1.00
*C* *‐ and* *X* *‐dependent dropout*								
D	0.00 (−0.33,0.33)	0.01 (−0.30,0.32)	0.05	0.02 (−1.38,1.43)	0.04	0.00 (−0.16,0.16)	0.00 (−0.36,0.37)	0.05
D(EM)	0.00 (−0.33,0.33)	0.01 (−0.30,0.32)	0.05	**1.03** (−0.43,2.49)	0.26	0.00 (−0.18,0.18)	**1.00** (0.58,1.43)	0.99
*Y* *‐dependent MNAR dropout*								
E	**−0.42** (−0.66,−0.17)	**0.12** (−0.09,0.33)	0.20	**0.71** (−0.12,1.55)	0.36	**−0.15** (−0.31,0.00)	**0.11** (−0.19,0.40)	0.10
E(EM)	**−0.31** (−0.56,−0.07)	**0.16** (−0.05,0.38)	0.32	**1.20** (0.32,2.07)	0.76	**−0.05** (−0.22,0.11)	**0.83** (0.47,1.19)	1.00
*U* *dependent MNAR dropout*								
F	0.00 (−0.3,0.29)	0.00 (−0.29,0.29)	0.06	0.01 (−1.15,1.17)	0.05	0.00 (−0.15,0.16)	0.00 (−0.32,0.32)	0.05
F(EM)	−0.01 (−0.31,0.3)	0.00 (−0.29,0.29)	0.06	**1.02** (−0.21,2.24)	0.37	0.00 (−0.17,0.17)	**1.00** (0.61,1.39)	1.00
*U* *‐ and* *X* *‐dependent MNAR dropout*								
G	**−0.52** (−0.82,−0.22)	0.06 (−0.22,0.34)	0.06	**0.56** (−0.61,1.73)	0.16	**−0.21** (−0.36,−0.05)	**0.09** (−0.23,0.42)	0.09
G(EM)	**−0.52** (−0.82,−0.22)	0.06 (−0.22,0.34)	0.06	**1.56** (0.32,2.79)	0.69	**−0.21** (−0.38,−0.04)	**1.09** (0.70,1.49)	1.00

In summary, this simulation shows that a variance difference across trial arms, in the observed data, at baseline, VD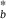, indicates outcome‐dependent MNAR dropout (scenario E), which may result in a biased CCA treatment effect estimate. If VD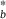 is zero but the variance difference across trial arms in the observed data at follow‐up, VD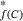, is nonzero, then one explanation is that dropout occurs according to the MNAR missingness mechanism in scenario G, which may result in a biased treatment effect estimate. Alternatively, this could be explained by effect modification together with a missingness mechanism that is MCAR (scenario A), MAR (scenarios B, C, and D), or MNAR (scenario F), which will result in an unbiased treatment effect estimate. If both VD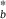 and VD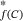 are zero, then the missingness mechanism is MCAR (scenario A), MAR (scenarios B, C, and D), or MNAR (scenario F) with no effect modification and the CCA treatment effect estimate will be unbiased. Adjusting for the outcome at baseline will result in an attenuation of the CCA estimator bias and a smaller VD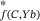, when dropout is MNAR as in scenarios E and G, irrespective of the presence of effect modification.

## 6 USING CONDITIONAL TRIAL ARM OUTCOME VARIANCES TO EVALUATE IMPUTATION MODELS

In this section, we consider the situation where there are measured covariates that are predictive of dropout and outcome, which are not included in the analysis model. In Section 3, we showed that for an MAR dropout mechanism, the outcome variances in the observed data are equal across trial arms, when conditioning on all variables that affect missingness. Additionally, we showed that this also holds when dropout is MNAR dependent on some unobserved variable, given that this variable is independent of treatment in the observed data. This property, in conjunction with the assumption of homogeneous treatment effects (assumption A1) and homoskedastic outcome errors (assumption A2), can be used to assess the plausibility of bias in a CCA analysis, by comparing the outcome variances across trial arms while conditioning on all analysis model variables. When data are missing, however, investigators may choose to use an MI approach, defining an imputation model that includes auxiliary variables that are not included in the main analysis model. In an MI model, assuming that dropout is MAR conditional on the imputation model variables and that the imputation model is correctly specified, we would expect the variance difference to be zero across the imputed datasets.

In this simulation study, we show that when dropout depends on some covariate, *C*_2_, and *X*, and *C*_2_ is excluded from the analysis model, the CCA treatment effect estimate is biased and there is an outcome variance difference across trial arms in the observed data. If *C*_2_ is included in the imputation model, however, fitting the same analysis model to the imputed datasets will result in an unbiased estimate of the treatment effect and no variance difference. Consequently, the outcome variance difference across trial arms in the imputed data can be used to assess the added value of including auxiliary variables in the imputation model.

### 6.1 Methods

**FIGURE 2:**
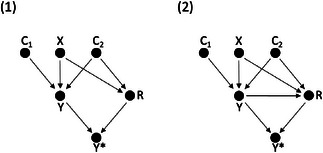
Two directed acyclic graphs (DAGs), depicting the relationship between binary treatment (*X*), continuous outcome (*Y*), two measured covariates (*C*_1_ and *C*_2_), and the response indicator (*R*). The observed outcome is some function f(Y,R) and denoted $Y^{*}$. (A) *Y* is MAR conditional on *X* and *C*_2_. (B) *Y* is MNAR, conditional on *X* and *C*_2_, with dropout dependent on *X*, *C*_2_, and *Y*.

We performed a simulation study with the outcome, *Y*, defined according to a linear model: $Y=\alpha +\beta X+\gamma_{1}C_{1}+\gamma_{2}C_{2}+\varepsilon_{Y}$, with *C*_1_ and *C*_2_ two observed independent continuous covariates, and the remaining terms defined as in (1) (Section 2). A total of N=1000 and N=10,000 patients were randomized to treatment, with a true treatment effect β=1, and trial arm outcome variances of 8. Dropout was simulated according to the two dropout mechanisms shown as DAGs in Figure 2, defined in the same manner as in Figure 1, with, for example, in DAG 1, *Y* affected by treatment, *X*, covariates *C*_1_ and *C*_2_, and outcomes MAR conditional on *X* and *C*_2_. Dropout was simulated under a logit mechanism, with 28% overall dropout.

In the observed data, we performed a CCA linear regression conditional on *C*_1_. Missing outcomes were imputed conditional on *C*_1_, *C*_2_, *X*, and $Y^{*}$, generating 10 complete datasets. Treatment effect estimates adjusted for *C*_1_ were obtained for each dataset and subsequently pooled using Rubin's rules (Rubin, 1996). The corresponding variance differences for both models were estimated conditional on *C*_1_. The 95% CIs were computed using the SD of the relevant estimate across simulations. We simulated 1000 datasets for each scenario, having verified, for each estimate, that the Monte Carlo SD and mean standard error were comparable, indicating that 1000 repetitions are sufficient. A more detailed description of the simulation framework, in accordance with ADEMP guidelines (Morris et al., 2019), is given in Online Appendix D.1.

### 6.2 Results

The simulation results for N=1000 are shown in Table 3. In scenario 1 (Figure 2a), dropout depends on *X* and *C*_2_, and, consequently, is MNAR conditional on analysis model covariates *C*_1_ and *X*, resulting in a biased CCA treatment effect estimate, with the corresponding outcome variance difference across trial arms nonzero. The dropout, however, is MAR conditional on *X* and *C*_2_, and fitting the same analysis model, which regresses *Y* on *X* and *C*_1_, to data imputed conditional on $Y^{*}$, *X*, *C*_1_, and *C*_2_, results in an unbiased treatment effect estimate and a nearzero variance difference. In scenario 2 (Figure 2b), dropout is a function of *Y*, in addition to *C*_2_ and *X*, and consequently, MNAR conditional on the analysis model covariates and also MNAR conditional on the imputation model covariates. This results in a biased CCA estimate in the observed data and a biased treatment effect estimate in the imputed data, with, for both, nonzero outcome variance differences across trial arms. In Online Appendix D.2, a companion table is provided for Table 3 (Table D.1), which includes results for a sample size of N=10,000 and various measures of simulation performance.

**TABLE 3:** Bias of the complete case analysis (CCA) and multiple imputation (MI) treatment effect estimates and outcome variance differences (VDs) across trial arms in the observed data, with 95% confidence intervals (CIs), for data (N=1000) simulated according to directed acyclic graphs (DAGs) 1 and 2 (Figure 2). (1) *Y* is a function of *C*_1_, *C*_2_, and *X*, with dropout dependent on *C*_2_ and *X*; (2) analogous to 1, with dropout additionally dependent on *Y*. Shown is the CCA treatment effect estimate, conditional on *C*_1_, with corresponding VD in the observed data (VD*), alongside the MI treatment effect estimate and VD (VD), estimated conditional on *C*_1_, with both *C*_1_ and *C*_2_ included in the imputation model. The estimates shown are mean values across 1000 simulated datasets of N=1000, with a true treatment effect β=1, and trial arm outcome variances of 8.

	Observed data		Imputed data	
	Bias (95% CI)	VD* (95% CI)	Bias (95% CI)	VD 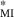 (95% CI)
1	−0.36 (−0.70,−0.01)	0.28 (−0.84,1.40)	−0.02 (−0.38,0.34)	0.08 (−0.85,1.01)
2	−0.56 (−0.84,−0.27)	0.72 (−0.08,1.53)	−0.48 (−0.75,−0.22)	0.75 (0.11,1.38)

Based on the outcome variance difference in the observed and imputed data, we would conclude, for scenario 1, that including variable *C*_2_ in the imputation model will result in a less biased estimate, while, for scenario 2, we would infer that the imputation model fails to address the dropout mechanism, suggesting that the data are MNAR given the variables in the imputation model.

Previously, in Section 3, we showed that if dropout depends on some covariate and *X*, and the two are not independent in the observed data, including the covariate in the analysis model will result in an unbiased CCA estimate and no outcome variance difference across trial arms in the observed data. Here, we show, when using MI, that if this covariate is omitted from the analysis model but included in the imputation model, the resulting treatment effect estimate will be unbiased and the outcome variance difference will be zero. Consequently, the outcome variance difference across trial arms in the imputed data can be used to assess the added value of including variables in the imputation model for explaining the missingness mechanism.

## 7 APPLICATION

### 7.1 An application using individual‐level data from the acupuncture trial

We now apply our method to individual‐level data from an RCT, which compared the effect of two treatments on 401 patients suffering from chronic headaches (Vickers et al., 2004; Vickers, 2006). The primary outcome was the headache score at 12 months, with higher values indicating worse symptoms. Patients were randomly allocated to acupuncture intervention (*N* = 205) or usual care (*N* = 196). The trial found a beneficial effect of acupuncture treatment, with a mean difference in headache score of −4.6 (95% CI: −7.0, −2.2), adjusted for baseline headache score and minimization variables age, sex, headache type, number of years of headache disorder, and site (general practices in England and Wales). At 12 months, 21% and 29% of patients in the acupuncture and usual care arm, respectively, had dropped out. The investigators noted that while dropouts were generally comparable across the two arms, their baseline headache score was on average higher.

Existing methods for assessing risk of bias due to dropout include checking if dropout is differential across trial arms (Higgins et al., 2012; Sterne et al., 2019), and if baseline covariate distributions are different across trial arms in patients who are still observed at the end of follow‐up (Groenwold et al., 2014). We assessed the relationship between trial arm and dropout by performing a logistic regression of the dropout indicator on treatment, which yielded an association of 0.38 (95% CI: −0.07, 0.84), with the CI just including the null. Note, however, that biased and unbiased treatment effect estimates can be obtained both when dropout is balanced and differential (Bell et al., 2013). We compared the baseline covariate distributions across trial arms by performing linear regressions of each covariate included in the primary analysis model on treatment, using the subset of patients still observed at 12 months, with the covariates standardized to facilitate comparisons. The largest point estimate and narrowest confidence interval were observed for the headache score at baseline (0.14, 95% CI: −0.09, 0.37), though the latter also included the null (Table 4). As for differential dropout, both MAR and MNAR dropout mechanisms can result in different baseline covariate distributions across trial arms, and consequently, neither method is a unique marker for the presence of MNAR dropout.

**TABLE 4:** Distribution of baseline variables (minimization variables and headache score at baseline) across trial arms in patients with observed values for the headache score at 12 months (161 and 140 patients in the acupuncture and usual care arm, respectively). Point estimates ($\hat{\beta }$) and 95% confidence intervals (CIs) were obtained in linear regressions of each standardized variable on treatment.

	$\hat{\beta }$	95% CI
Age	0.02	(−0.21, 0.25)
Sex	−0.08	(−0.31, 0.14)
Headache type	0.01	(−0.22, 0.23)
Years of headache disorder	−0.04	(−0.27, 0.19)
Site	−0.01	(−0.24, 0.22)
Baseline headache score	−0.14	(−0.37, 0.09)

Using the outcome variance differences across trial arms at 12 months (VD_12_) and at baseline (VD_*b*_), we assessed the risk of bias due to MNAR dropout for an unadjusted CCA model (M1), a model adjusted for the minimization variables (M2), and a model adjusted for the minimization variables and baseline headache score (M3). VD_12_ and VD_*b*_ were estimated using the studentized Breusch–Pagan test, for the subset of patients still observed at 12 months. Results are reported in Table 5.

**TABLE 5:** Complete case analysis (CCA) treatment effect estimates ($\hat{\beta }$) and outcome variance differences (VDs) across trial arms, with 95% confidence intervals (CIs), for data from the acupuncture randomized controlled trial (RCT), which compared the effect of a treatment policy using acupuncture versus usual care on headache scores at 12 months follow‐up. VDs were calculated at baseline (VD_*b*_) and at 12 months (VD_12_), for the set of patients still observed at 12 months. M1) Headache score is regressed on treatment; M2) Regression is adjusted for five minimization variables; M3) Regression is adjusted for five minimization variables and the baseline headache score (analysis model used in the published trial results).

	$\hat{\beta }$	95% CI	VD_*b*_	95% CI	VD_12_	95% CI
M1	−6.1	(−9.58,−2.61)	−81.46	(−183.21,20.30)	−100.28	(−222.00,21.44)
M2	−6.07	(−9.54,−2.60)			−99.08	(−220.88,22.71)
M3	−4.64	(−7.08,−2.19)			21.23	(−26.83,69.30)

The unadjusted model (M1), regressing headache score on treatment, showed a beneficial effect of acupuncture therapy (−6.1, 95% CI: −9.6, −2.6). At 12 months, the outcome variances in the acupuncture arm and usual care arm were 188.2 and 289.4, respectively, with a variance difference, VD_12_=‐100.3 (95% CI: −222.0, 21.4). This result is compatible with a large negative variance difference (−222.0), with a smaller outcome variance in the acupuncture arm, and a small positive variance difference (21.4), with a smaller outcome variance in the usual care arm. At baseline, we estimated an outcome variance difference of VD_*b*_ = −81.5 (95% CI: −183.2, 20.3), which is once again compatible with a large negative variance difference and a small positive variance difference. A substantial outcome variance difference at baseline raises concerns of outcome‐dependent MNAR dropout, whereas, at follow‐up, an outcome variance difference may have multiple causes: MNAR dropout, heteroskedastic errors, or treatment effect heterogeneity resulting from effect modification. Adjusting for the five minimization variables (M2) did not affect the estimated treatment effect or VD_12_, whereas additionally adjusting for baseline headache score (M3) resulted in an attenuated treatment effect of −4.64 (95% CI: −7.08, −2.19) and a greatly reduced positive VD_12_ of 21.23 (95% CI: −26.83, 69.30), with much tighter confidence bounds. In Section 5.3, we showed that when dropout is MNAR, conditioning on the outcome at baseline results in a smaller outcome variance difference at follow‐up and attenuation of the CCA estimator bias.

However, in the event that the outcome variance difference at follow‐up is the result of treatment effect heterogeneity, conditioning on an effect modifier will also result in a decreased variance difference (Mills et al., 2021). A simple way to check if a variable is an effect modifier is to perform a stratified analysis. We repeated the regression of M3 (Table 5) in patients with baseline headache scores below the mean, and in patients with scores above the mean, yielding estimates of −2.92 (95% CI: −5.15, −0.69) and −6.66 (95% CI: −12.49, −0.83), respectively. The difference in treatment effect estimate in patients with lower and higher baseline scores suggests that the headache score at baseline may act as an effect modifier. For comparison, we performed an analogous analysis dividing the patients according to age, which showed no difference in treatment effect estimate between patients below mean age (−4.76; 95% CI: −8.89, −0.63) and above mean age (−4.57; 95% CI: −7.73, −1.40). This suggests that the baseline headache score may be acting as an effect modifier, which would imply that the observed outcome variance difference at 12 months may at least in part be the result of effect modification in the intervention arm.

An outcome variance difference at follow‐up may also result from the presence of heteroskedastic outcome errors. This can be assessed by checking if variability in the outcome at baseline is different for patients with lower and higher values. We did this by ordering the baseline headache score values and dividing them into six bins. The variances across bins showed no evidence against homoskedastic outcome errors, with no corresponding increase or decrease in variance observed (Table E.1, Online Appendix E).

In summary, our results suggest that the CCA estimate, adjusted for minimization variables and baseline headache score may be biased due to outcome‐dependent dropout, and that the true treatment effect may be more modest. The magnitude of this bias is, however, likely partly reduced by conditioning on the baseline headache score. The bias can be expected to be further attenuated when conditioning on additional variables that are predictors of the outcome, either by including them in the main analysis model or, if using an MI approach, in the imputation model. In the original trial publication (Vickers et al., 2004), the authors additionally obtained the treatment effect estimate when imputing the 12‐month dropouts using auxiliary variables that were highly correlated with the headache score at 12 months, including the headache score measured at a previous time point (3 months) and a post‐hoc global assessment of headache severity. This yielded a smaller treatment effect estimate of −3.9 (95% CI: −6.4, −1.4), when compared to the CCA estimate adjusted for minimization variables and baseline headache score (−4.6; 95% CI: −7.1, −2.2). This attenuation on imputing the missing 12‐month outcomes using variables highly correlated with the outcome further supports our conclusion that the CCA treatment effect estimate is likely affected by outcome‐dependent dropout. This can be further investigated by performing sensitivity analyses under assumption of a dropout mechanisms that is MNAR dependent on the outcome.

Note that observing a variance difference at baseline is sufficient to raise concern of outcome‐dependent dropout. Further investigating the variance difference at follow‐up, as we do here, is then not strictly necessary, though it may nevertheless be interesting for interpretation purposes. Investigating the possibility of heterogeneous treatment effects and heteroscedastic errors, however, becomes necessary when there is a non‐outcome dependent MNAR dropout mechanism, which will only result in an outcome variance difference across trial arms at follow‐up but not at baseline.

### 7.2 An application using summary‐level data from the POPPI trial

The POPPI trial investigated whether a preventive, complex psychological intervention, initiated in the intensive care unit (ICU), would reduce the development of subsequent posttraumatic stress disorder (PTSD) symptoms at 6 months in ICU patients (Wade et al., 2019). Symptom severity was quantified using the PTSD symptom scale‐self‐report (PSS‐SR) questionnaire, with higher values indicating greater severity. Twenty‐four ICUs were randomized to intervention or control, with intervention ICUs providing usual care during a baseline period and the preventive intervention during the intervention period, and control ICUs providing usual care throughout. At 6 months follow‐up, 79.3% of patients had completed the PSS‐SR questionnaire, with no difference across study arms. The trial found no beneficial effect of intervention, with a mean difference in PSS‐SR score of −0.03 (95% CI: −2.58, 2.52), adjusted for age, sex, race/ethnicity, deprivation, preexisting anxiety/depression, planned admission following elective surgery, and the Intensive Care National Audit & Research Centre (ICNARC) Physiology Score.

Using summary statistics from the published study, we performed a *t*‐test for the variance difference (Mills et al., 2021) between trial arms at 6 months, to assess if the study's reported null result may have been biased by MNAR dropout. Published estimates were means with 95% CIs, adjusted for the previously listed variables, which we used to calculate the outcome variances and corresponding variance differences). We found no evidence for a variance difference across trial arms at baseline (VD_*b*_ = 11.2; 95% CI: −22.7, 45.2), but a greater variance in the intervention arm at 6 months (VD_6_ = 52.5; 95% CI: 18.8, 86.2).

No variance difference across trial arms in the outcome at baseline indicates that dropout is not outcome‐dependent, whereas the variance difference at follow‐up may be the result of treatment effect heterogeneity, heteroskedastic outcome errors, or MNAR dropout that does not depend on outcome, as in scenario of Figure 1(g), where dropout depends on some unobserved covariate, *U*, and treatment. For nonoutcome‐dependent MNAR dropout to result in bias and a variance difference, this requires  *U* to interact with treatment in the dropout mechanism, which will result in differential dropout across trial arms. In the POPPI trial data, however, dropout was balanced at 6 months follow‐up, suggesting that nonoutcome‐dependent MNAR dropout is unlikely to be the cause of the observed outcome variance difference across trial arms at follow‐up. An outcome variance difference at follow‐up may also be the result of heteroskedastic outcome errors. This, however, requires that a treatment effect be present, whereas, here, a null treatment effect was estimated. In summary, our results suggest that there is no MNAR dropout in the POPPI trial at 6 months follow‐up, but that there may be treatment effect heterogeneity, which, on average, results in a null treatment effect. Further investigation into potential effect modifiers would require access to individual‐level data.

## 8 DISCUSSION

In this paper, we show that, in RCTs, the outcome variance difference across trial arms at baseline, in the set of study participants who did not drop out, is an indicator of outcome‐dependent MNAR dropout, and consequently, a biased CCA treatment effect estimate. In contrast, when outcomes are MAR or MCAR, this baseline variance difference will be zero. We also show that the outcome variance difference across trial arms in the observed data at follow‐up can be an indicator of both outcome‐dependent MNAR dropout and nonoutcome dependent MNAR dropout, both of which may result in a biased treatment effect estimate. A variance difference across trial arms at follow‐up, in the observed data, however, can only be meaningfully interpreted when the outcome variances can be expected to be equal across trial arms in the full data. This requires two assumptions: first, that there is no treatment effect heterogeneity; second, that the errors of the outcome are homoskedastic.

Treatment effect heterogeneity can be thought of as nonrandom variability in treatment response that is attributable to patient characteristics. How plausible it is that heterogeneity is absent depends strongly on intervention type and study population. Efficacy trials, for example, typically have stricter inclusion criteria, resulting in more homogeneous study populations, and are less prone to large variations in treatment response. In contrast, pragmatic trials with broad eligibility criteria are more likely to have heterogeneous treatment effects (Varadhan & Seeger, 2013). As treatment effect heterogeneity affects the outcome variance of the intervention arm (Mills et al., 2021), it is a second potential cause of observed outcome variance differences across trial arms. The presence of effect modification can be investigated by performing a stratified analysis, where the effect modifier serves as a stratification variable (Corraini et al., 2017). Alternatively, if the observed difference in outcome variance between trial arms is solely the result of effect modification, then conditioning on the effect modifier and the interaction term between effect modifier and trial arm can be expected to remove all evidence of a variance difference.

An outcome variance difference across trial arms may also result from heteroskedastic outcome errors. Specifically, if the variance of the outcome errors is related to the outcome and if treatment has a causal effect on the outcome, this may cause a difference in outcome variances across trial arms at follow‐up. For example, suppose an outcome such as body mass index (BMI) has greater day‐to‐day variability in people with higher BMI. Then, if the treatment lowers the mean BMI in the intervention arm, this will result in a comparatively smaller intervention arm variance. A simple way to investigate this is to consider the outcome variable measured at baseline, group its values into bins, and establish if the outcome error variance is different in bins with higher and lower mean values.

We additionally propose employing the (conditional) outcome variance difference across trial arms in the observed data as an MNAR bias assessment tool, and, indirectly, as a model building tool, which can be used to assess the added value of including variables for explaining the missingness mechanism. This method is easily implemented, using existing tests available in standard software, and has a straightforward interpretation of results. In Section 7, we demonstrated how outcome variance differences can be used to assess the risk of MNAR bias for various models, using both individual‐level data from the acupuncture trial, and summary‐level data from the POPPI trial.

The outcome variance difference across trial arms at baseline and at follow‐up is suitable for assessing the risk of dropout bias for analysis models that are estimated with OLS linear regression, and assume that the outcome is continuous and given by a linear model. These methods cannot be used for noncontinuous outcomes, such as binary or time‐to‐event outcomes.

A second limitation of our proposed method is its comparatively modest power, with the power to detect an outcome variance difference lower than the power to detect a difference in outcome means. Brookes et al. (2004) showed that if a trial is powered to detect a mean difference of a given size, then in order to detect an interaction effect of the same magnitude, the sample size needs to be approximately four times larger. Mills et al. (2021) found comparable numbers for the power to detect a variance difference.

Instead of using our method as a strict significance test with a dichotomous conclusion, we recommend assessing the practical implications of the values inside the confidence interval (Amrhein et al., 2019; Andrade, 2019; Wasserstein et al., 2019). For example, in the individual‐level data application of Section 7, at follow‐up, the outcome variances in the observed data were 188.2 and 289.4, in the intervention usual care arm, respectively, with a variance difference of −100.3 (95% CI: −222.0, 21.4). At baseline, in the observed data, we estimated a variance difference of −81.46 between trial arms, with a 95% CI of −183.21–20.30. These results are compatible with a large negative outcome variance difference as well as a small positive outcome variance difference. These large negative variance difference would raise concerns of MNAR dropout, with the large negative outcome variance difference at baseline specifically suggestive of outcome‐dependent dropout.

While a variance difference across trial arms in the observed data at baseline indicates outcome‐dependent MNAR dropout, interpreting a variance difference at follow‐up is less straightforward. For the latter, we suggest performing further analyses to identify the presence of heteroskedastic outcome errors and treatment effect heterogeneity, and using expert and contextual knowledge. If the presence of both heteroskedastic errors and treatment effect heterogeneity is judged to be unlikely, then the possibility of nonoutcome‐dependent MNAR dropout should be investigated, for example, by conditioning on additional covariates in the analysis model or, if using MI, in the imputation model, and assessing the effect of this on the variance difference at follow‐up. If the results suggest that dropout may be MNAR and that, consequently, the treatment effect estimate is at risk of bias, this motivates performing a sensitivity analysis to assess the robustness of the main analysis results under a plausible MNAR assumption. For example, we may observe a variance difference across trial arms in the observed data at baseline, suggesting outcome‐dependent dropout, and also observe that the treatment effect estimate becomes smaller if we additionally condition on covariates that are correlated with the outcome. This would suggest outcome‐dependent dropout that results in overestimation of the treatment effect, which may occur, for example, if, on average, more poor responders drop out. A natural subsequent step would involve performing a sensitivity analysis under the MNAR assumption of worse value dropout and investigating how strong this mechanism must be for the material conclusion to be affected.

## CONFLICT OF INTEREST STATEMENT

The authors have declared no conflict of interest.

### OPEN RESEARCH BADGES

This article has earned an Open Data badge for making publicly available the digitally‐shareable data necessary to reproduce the reported results. The data is available in the Supporting Information section.

This article has earned an open data badge “**Reproducible Research**” for making publicly available the code necessary to reproduce the reported results. The results reported in this article could fully be reproduced.

## Supporting information

Supporting Information

## Data Availability

Individual‐level patient data from the acupuncture trial (ISRCTN96537534) are publicly available and can be found in the supplementary materials of Vickers 2006. This trial was approved by the South West Multicentre Research Ethics Committee and appropriate local ethics committees.
