# Supplementary material for: Trial arm outcome variance difference after dropout as an indicator of missing-not-at-random bias in randomized controlled trials
Source: Biom J. Author manuscript; Available in PMC 2024 Jan 15. (PMC7615524; doi:10.1002/bimj.202200116)
Supplement: Appendix [file EMS193239-supplement-Appendix.pdf]

# Appendices to 'Trial arm outcome variance difference after dropout as an indicator of missing-not-at-random bias in randomised controlled trials'

Appendix A provides the detailed proofs of Section 3 of the main text. Appendix B derives equations (19), (20), and (22) in Section 5.1 of the main text. Appendix C and D provide additional details and results for the simulation studies described in Sections 5 and 6 of the main text, respectively. Appendix E contains Supplementary Table E.1, which is referred to in Section 7.1 of the main text.

## A MAR and MNAR dropout and outcome variances across trial arms in the observed data (Section 3)

This appendix provides the proof for Proposition 1 and Proposition 2 of Section 3 of the main text in detail. We reiterate the key elements of Section 2 (Notation) for ease of reading.

In an RCT, the outcome variances across trial arms can be expected to be the same across follow-up, given that the treatment effects are homogeneous and the outcome errors homoskedastic. We reiterate these two assumptions here:

**Assumption A1** *There is no treatment effect heterogeneity so that the treatment effect, $\beta$, is the same for every individual.*

**Assumption A2** *The errors are homoscedastic so that the error term of the outcome (A.1), $\varepsilon_Y$, does not depend on treatment or on $Y$ itself.*

Let the outcome, $Y$, be defined as in equation (1) of the main text (Section 2):

$$Y = \alpha + \beta X + \gamma U + \delta C + \varepsilon_Y, \tag{A.1}$$

with $\alpha$ some intercept, $\beta$ the treatment effect, $\gamma$ and $\delta$ the effects of $U$ and $C$ on $Y$, respectively, $\varepsilon_Y$ the mean-zero error term, with $\varepsilon_Y$ independent of $X$, $U$, and $C$, and with $U$ and $C$ additionally independent of $X$. Let $R$ denote the response indicator, with $R = 1$ when $Y$ is observed, and $R = 0$ when $Y$ is missing, and let $Y^*$ denote the outcome in the observed data.

Let $\beta^*$ denote the unconditional treatment effect in the observed data:

$$\beta^* = \mathbb{E}[Y|X=1,R=1] - \mathbb{E}[Y|X=0,R=1],$$

with the bias, $B$, of the CCA treatment effect estimate given by the difference of the estimate in the observed and full data:

$$B = \beta^* - \beta, \tag{A.2}$$

and the outcome variance difference across trial arms in the observed data given by:

$$\mathrm{VD}^* = \mathrm{var}(Y|X=1,R=1) - \mathrm{var}(Y|X=1,R=1). \tag{A.3}$$

Let $\beta^*_C$ denote the treatment effect in the observed data, conditional on covariate, $C$:

$$\beta^*_C = \mathbb{E}[Y|X=1,C,R=1] - \mathbb{E}[Y|X=0,C,R=1], \tag{A.4}$$

with corresponding bias

$$B_C = \beta^*_C - \beta, \tag{A.5}$$

and outcome variance difference across trial arms

$$\mathrm{VD}^*_C = \mathrm{var}(Y|X=1,C,R=1) - \mathrm{var}(Y|X=0,C,R=1). \tag{A.6}$$

We are interested in the bias of the treatment effect estimate, $B_C$ (A.5), when the treatment effect, $\beta^*_C$ (A.4), is estimated while adjusting for some observed baseline covariate, $C$. Analogously, we define the variance difference as the difference in trial arm outcome variances, conditional on $C$, denoted $\mathrm{VD_C}$ (equation (12) of the main text) and $\mathrm{VD}^*_C$ (A.6) in the full and observed data, respectively.

## A.1 Proposition 1

Let $\mathsf{P}(Y|X,C)$ denote the density of $Y$, conditional on $X$ and some observed baseline covariate, $C$, in the full data, and $\mathsf{P}(Y|X,C,R=1)$ the corresponding density in the observed data.

**Proposition 1** *The densities $\mathsf{P}(Y|X,C)$ and $\mathsf{P}(Y|X,C,R)$ will be identical only when dropout is MCAR or MAR, with $R$ independent of $Y$ given the variables included in the analysis model ($R \perp\!\!\!\perp Y|X,C$). Any quantities derived from the densities, such as the mean difference and variance difference across $X$, will be also the same. If assumptions A1 and A2 are satisfied so that the variances of the outcome in the two trial arms are equal in the full data, then $\mathsf{P}(Y|X,C)$=$\mathsf{P}(Y|X,C,R)$ implies that the variances of the outcome in the two trial arms are also equal in the observed data.*

Figure A.1.A depicts a MCAR dropout mechanism, with a response indicator, $R$, unaffected by any observed or unobserved variables. Then, $Y$ is unconditionally independent from $R$ ($Y \perp\!\!\!\perp R$) so that $\mathsf{P}(Y|X,C) = \mathsf{P}(Y|X,C,R=1)$. Figures A.1.B, C and D depict MAR dropout mechanisms, with dropout dependent on treatment, $X$, on some baseline covariate, $C$, and on both $X$ and $C$, respectively. In Figure A.1.B, $R$ depends on $X$ only

so that $X$ directionally separates (d-separates) $Y$ from $R$, which implies the conditional independence $Y \perp\!\!\!\perp R|X$. As we already condition on $X$ in $\mathsf{P}(Y|X,C)$, it is once again equal to $\mathsf{P}(Y|X,C,R=1)$. Analogously, for $C$-dependent dropout in Figure A.1.C, $Y \perp\!\!\!\perp R|C$, and, for dropout dependent on $C$ and $X$ in Figure A.1.D, $Y \perp\!\!\!\perp R|(X,C)$, with for both $\mathsf{P}(Y|X,C) = \mathsf{P}(Y|X,C,R=1)$.

Consequently, if dropout is MAR given $X$ and $C$, the density of $Y$, conditional on $X$ and $C$, will be the same in the full and observed data. This implies that the variances of $Y$ in the observed data in $X=0$ and $X=1$ will be equal in expectation, given that the variances in the full data are. The latter requires that there is no treatment effect heterogeneity (assumption A1) and that the outcome errors are homoscedastic (assumption A2).

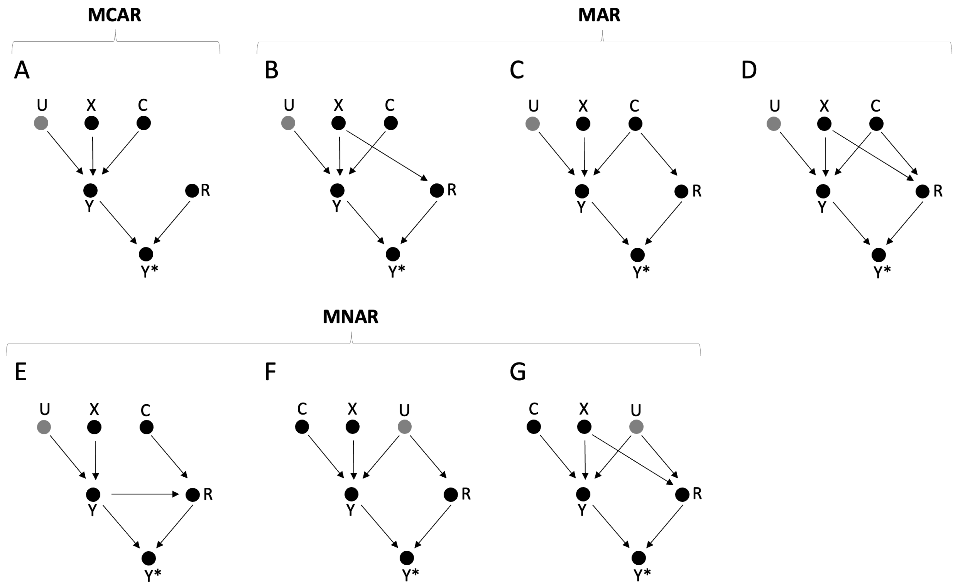


**Figure A.1:** Seven directed acyclic graphs (DAGs), depicting the relationship between binary treatment ($X$), continuous outcome ($Y$), an unmeasured continuous variable ($U$), a measured continuous variable ($C$) and the response indicator ($R$). The observed outcome is some function $f(Y,R)$ and denoted $Y^*$. The dropout mechanisms —missing completely at random (MCAR), missing at random (MAR) and missing not at random (MNAR)— are defined with respect to an analysis model which regresses the observed outcome, $Y^*$ on $X$ and $C$.

Figures A.1.E, F and G depict MNAR dropout mechanisms, with dropout dependent on outcome, on some unobserved covariate, and on treatment and some unobserved covariate both.

In Figure A.1.E dropout is dependent on the outcome $Y$. By definition, we can write the joint density of $Y$, $X$, and $C$ as

$$\mathsf{P}(Y, X, C) = \mathsf{P}(Y|X, C)\mathsf{P}(X, C). \tag{A.7}$$

$Y$ d-separates $X$ and $C$ from $R$, implying the conditional independence $(X, C) \perp\!\!\!\perp R|Y$ so that

$$\mathsf{P}(X, C, R|Y) = \mathsf{P}(X, C|Y)\mathsf{P}(R|Y).$$

This can be rewritten as

$$\mathsf{P}(X, C, R, Y) = \mathsf{P}(Y, X, C)\mathsf{P}(R|Y),$$

and

$$\mathsf{P}(Y, X, C) = \frac{\mathsf{P}(X, C, R, Y)}{\mathsf{P}(R|Y)} = \mathsf{P}(Y|X, C, R)\frac{\mathsf{P}(R|X, C)}{\mathsf{P}(R|Y)}\mathsf{P}(X, C). \tag{A.8}$$

From (A.7) and (A.8) we note that

$$\mathsf{P}(Y|X, C) = \mathsf{P}(Y|X, C, R)\frac{\mathsf{P}(R|X, C)}{\mathsf{P}(R|Y)},$$

and that, under a $Y$-dependent MNAR dropout mechanism, the density of $Y$, conditional on $X$ and $C$ in the full data ($\mathsf{P}(Y|X, C)$) is not equal to the corresponding density in the observed data ($\mathsf{P}(Y|X, C, R = 1)$). As the densities are different in the observed and full data, this implies that the means and variances can be different also.

In Figure A.1.F, dropout depends on unobserved variable $U$, and in Figure A.1.G, dropout depends on $U$ and treatment $X$, implying the conditional independence $Y \perp\!\!\!\perp R|U$ and $Y \perp\!\!\!\perp R|X, U$, respectively. While $\mathsf{P}(Y|X, C, U) = \mathsf{P}(Y|X, C, U, R = 1)$, we cannot condition on $U$ and can only estimate quantities derived from $\mathsf{P}(Y|X, C, R = 1)$, which is not equal to $\mathsf{P}(Y|X, C)$, as conditioning on $X$ and $C$ is insufficient to block the path from $Y$ to $R$.

## A.2 Proposition 2

We define the outcome, $Y$, be defined as in (A.1), with the intervention arm mean given by:

$$\mu_1 = \mathbb{E}[Y|X = 1] = \alpha + \beta + \gamma\mathbb{E}[U|X = 1] + \delta\mathbb{E}[C|X = 1] + \mathbb{E}[\varepsilon_Y|X = 1], \tag{A.9}$$

and the comparator arm mean given by

$$\mu_0 = \mathbb{E}[Y|X = 0] = \alpha + \gamma\mathbb{E}[U|X = 0] + \delta\mathbb{E}[C|X = 0] + \mathbb{E}[\varepsilon_Y|X = 0]. \tag{A.10}$$

The variance of $Y$ (A.1), in the full data, for a given trial arm, $j$, is given by:

$$\begin{aligned} \mathrm{var}(Y|X = j) =& \gamma^2\mathrm{var}(U|X = j) + \delta^2\mathrm{var}(C|X = j) + \\ & 2\gamma\delta\mathrm{cov}(U, C|X = j) + \mathrm{var}(\varepsilon_Y|X = j), \end{aligned} \tag{A.11}$$

.

and in the observed data by:

$$\begin{aligned}\text{var}(Y|X=j,R=1)=&\gamma^2\text{var}(U|X=j,R=1)+\delta^2\text{var}(C|X=j,R=1)+\\&\text{var}(\varepsilon_Y|X=j,R=1)+2\gamma\text{cov}(U,\varepsilon_Y|X=j,R=1)+\\&2\delta\text{cov}(C,\varepsilon_Y|X=j,R=1)+2\gamma\delta\text{cov}(U,C|X=j,R=1).\end{aligned} \tag{A.12}$$

.

**Proposition 2** *Let the outcome, $Y$, be defined as in (A.1). If dropout depends on unmeasured covariate, $U$, and if $(U \perp\!\!\!\perp X)|R$, then the outcome mean difference and variance difference across trial arms in the full data can be estimated from the observed data, without conditioning on $U$. If assumptions A1 and A2 are satisfied so that the the variances of the outcome in the two trial arms are equal in the full data, this implies that the variances of the outcome in the two trial arms are also equal in the observed data.*

For all scenarios in Figure A.1, we assume that the measured and unmeasured covariates are independent of treatment ($C \perp\!\!\!\perp X$ and $U \perp\!\!\!\perp X$), which can be expected to hold in a randomised trial setting. Under the additional assumption of homoscedastic errors (assumption A2), $\varepsilon_Y \perp\!\!\!\perp X$. Consequently, $\mathbb{E}[U|X=0]=\mathbb{E}[U|X=1]$, $\mathbb{E}[C|X=0]=\mathbb{E}[C|X=1]$, and $\mathbb{E}[\varepsilon_Y|X=0]=\mathbb{E}[\varepsilon_Y|X=1]$ so that the treatment effect in the full data, $\beta$, is given by the unconditional difference between the intervention arm mean (A.9) and comparator arm mean (A.10), without the need for conditioning on $U$ or $C$. If these independencies also hold in the observed data so that $U \perp\!\!\!\perp X|R$, $C \perp\!\!\!\perp X|R$, and $\varepsilon_Y \perp\!\!\!\perp X|R$, then the unconditional treatment effect estimate in the observed data, $\beta^*=\beta$, and the bias of the CCA treatment effect estimate, $B=0$ (A.2).

In Figure A.1.F, dropout depends on unmeasured variable $U$. While this is an MNAR dropout mechanism, it results in an unbiased CCA treatment effect estimate, as $U$ is independent of $X$ in the observed data ($U \perp\!\!\!\perp X|R$) so that $\mathbb{E}[U|X=0,R=1]=\mathbb{E}[U|X=1,R=1]$. The same reasoning can be applied to Figure A.1.G, where dropout is MNAR and depends on $X$ and $U$. This, however, requires the additional assumption that the effects of $X$ and $U$ on $R$ are independent. For the purposes of this paper, we do not make this assumption, and allow $X$ and $U$ to interact so that, in the observed data, $X$ and $U$ are no longer independent ($X \not\perp\!\!\!\perp U|R$). If, additionally $U$ is also related to $C$, then $C$ and $U$ are also no longer independent in the observed data ($X \not\perp\!\!\!\perp C|R$). Then, in Figure A.1.G, $\mathbb{E}[U|X=0,R=1]\neq\mathbb{E}[U|X=1,R=1]$ and $\mathbb{E}[C|X=0,R=1]\neq\mathbb{E}[C|X=1,R=1]$, and the treatment effect estimate is given by

$$\begin{aligned}\beta^*=&\beta+\gamma(\mathbb{E}[U|X=1,R=1]-\mathbb{E}[U|X=0,R=1])+\\&\delta(\mathbb{E}[C|X=1,R=1]-\mathbb{E}[C|X=0,R=1]),\end{aligned} \tag{A.13}$$

with a CCA estimator bias of

$$\begin{aligned}B=\beta^*-\beta=&\gamma(\mathbb{E}[U|X=1,R=1]-\mathbb{E}[U|X=0,R=1])+\\&\delta(\mathbb{E}[C|X=1,R=1]-\mathbb{E}[C|X=0,R=1]).\end{aligned} \tag{A.14}$$

If we additionally condition on $C$, then the expected bias of the treatment effect estimate, $\beta^*_C$, is given by

$$B_C = \beta_C^* - \beta = \gamma(\mathbb{E}[U|X=1,C,R=1] - \mathbb{E}[U|X=0,C,R=1]). \tag{A.15}$$

Note that if $U \perp\!\!\!\perp C$, (A.14) and (A.15) will both simplify to

$$B = B_C = \gamma(\mathbb{E}[U|X=1,R=1] - \mathbb{E}[U|X=0,R=1]).$$

Under the linear model of $Y$ in (A.1), $U \perp\!\!\!\perp X$, $C \perp\!\!\!\perp X$, $\varepsilon_Y \perp\!\!\!\perp X$ (assumption A2). If we additionally assume that the treatment effect are homogeneous (assumption A1), then, in the full data, all variance components in (A.11) will be equal across trial arms (e.g., $\text{var}(U|X=0) = \text{var}(U|X=1)$) so that the outcome variance difference across trial arms, in the full data, $\text{VD} = \text{var}(Y|X=1) - \text{var}(Y|X=0) = 0$. Once again, in the observed data, these independencies hold for $U$-dependent (Figure A.1.F) dropout, but not for $U$- and $X$-dependent dropout (Figure A.1.G). When dropout depends on $U$ and $X$, in such a way that $U$ is not independent of $X$ in the observed data ($U \not\perp\!\!\!\perp X|R$), then, in (A.12), $\text{var}(U|X=0,R=1) \neq \text{var}(U|X=1,R=1)$. If, additionally, $C$ and $U$ are related, then also $C \not\perp\!\!\!\perp X|R$ and, consequently, $\text{var}(C|X=0,R=1) \neq \text{var}(C|X=1,R=1)$ and $\text{cov}(U,C|X=0,R=1) \neq \text{cov}(U,C|X=1,R=1)$ The variance difference across trial arms in the observed data, $\text{VD}^*$, is then given by,

$$\begin{aligned}\text{VD}^* =& \text{var}(Y|X=1,R=1) - \text{var}(Y|X=0,R=1) = \\ & \gamma^2\big(\text{var}(U|X=1,R=1) - \text{var}(U|X=0,R=1)\big) + \\ & \delta^2\big(\text{var}(C|X=1,R=1) - \text{var}(C|X=0,R=1)\big) + \\ & 2\gamma\delta\big(\text{cov}(U,C|X=1,R=1) - \text{cov}(U,C|X=1,R=1)\big).\end{aligned} \tag{A.16}$$

.

If we additionally condition on $C$, then the variance difference across trial arms in the observed data, $\text{VD}_C^*$, is given by

$$\begin{aligned}\text{VD}_C^* =& \text{var}(Y|X=1,C,R=1) - \text{var}(Y|X=0,C,R=1) = \\ & \gamma^2\big(\text{var}(U|X=1,C,R=1) - \text{var}(U|X=0,C,R=1)\big).\end{aligned} \tag{A.17}$$

.

Note that if $C \perp\!\!\!\perp U$, (A.16) and (A.17) both reduce to $\text{VD}^* = \text{VD}_C^* = \gamma^2\big(\text{var}(U|X=1,R=1) - \text{var}(U|X=0,R=1)\big)$.

Table 1 in the main text provides an overview of when the seven dropout scenarios of Figure A.1 (Figure 1 in the main text) result in a biased CCA estimate and an outcome variance difference across trial arms in the observed data, for a linear regression of $Y$ on $X$ and $C$.

# B Variance formulae, Section 5

This appendix provides more detailed derivations for the outcome variance difference across trial arms at baseline and at follow-up when assumption A1 is violated and the treatment

effects are heterogeneous (Section B.1), and when assumption A2 is violated and the outcome errors are heteroscedastic (Section B.2).

## B.1 Heterogeneous treatment effects (assumption 1 is violated)

Here, we show that when assumption A1 is violated, resulting in heterogeneous treatment effects, the outcome variances in the full data are different across trial arms. Let the outcome at follow-up, $Y_f$ be defined according to equation (17) of the main text, with $X$ and $\varepsilon_f$ independent of each other and independent of $C$, $U$, $S$, with $S$ independent of $C$ and $U$, and with $\varepsilon_f$ and $\varepsilon_b$ correlated. Below, we derive equations (19), (20), and (21) of the main text. By definition, the variance of $Y_f$ in a the comparator arm ($X = 0$) is given by:

$$\begin{aligned}\text{var}(Y_f|X=0) =& \gamma_b^2\text{var}(U|X=0) + \delta_b^2\text{var}(C|X=0) + \text{var}(\varepsilon_b|X=0)+\\ & 2\gamma_b\delta_b\text{cov}(U,C|X=j) + 2\gamma_b\text{cov}(U,\varepsilon_f|X=0) + 2\delta_b\text{cov}(C,\varepsilon_f|X=0)+\\ & 2\gamma_b\gamma\text{cov}(U,U|X=0) + 2\gamma_b\delta\text{cov}(U,C|X=0) + 2\gamma_b\text{cov}(U,\varepsilon_f|X=0)+\\ & 2\delta_b\delta\text{cov}(C,C|X=0) + 2\delta_b\text{cov}(C,\varepsilon_f|X=0) + 2\text{cov}(\varepsilon_b,\varepsilon_f|X=0)+\\ & \gamma^2\text{var}(U|X=0) + \delta^2\text{var}(C|X=0) + \text{var}(\varepsilon_f|X=0)+\\ & 2\gamma\delta\text{cov}(U,C|X=0) + 2\gamma\text{cov}(U,\varepsilon_f|X=0) + 2\delta\text{cov}(C,\varepsilon_f|X=0).\end{aligned} \tag{B.1}$$

With $C \perp\!\!\!\perp \varepsilon_f$ and $U \perp\!\!\!\perp \varepsilon_f$, the corresponding covariance terms drop out, and (B.1) reduces to

$$\begin{aligned}\text{var}(Y_f|X=0) =& \gamma_b^2\text{var}(U|X=0) + \delta_b^2\text{var}(C|X=0) + \text{var}(\varepsilon_b|X=0)+\\ & 2\gamma_b\delta_b\text{cov}(U,C|X=0) + 2\gamma_b\delta\text{cov}(U,C|X=0) + 2\gamma\delta_b\text{cov}(U,C|X=0)+\\ & 2\gamma_b\gamma\text{cov}(U,U|X=0) + 2\delta_b\delta\text{cov}(C,C|X=0) + 2\text{cov}(\varepsilon_b,\varepsilon_f|X=0)+\\ & \gamma^2\text{var}(U|X=0) + \delta^2\text{var}(C|X=0) + \text{var}(\varepsilon_f|X=0)+\\ & 2\gamma\delta\text{cov}(U,C|X=0).\end{aligned} \tag{B.2}$$

As the covariance of a variable with itself is equal to the variance, (B.2) simplifies further to (equation (19) of the main text)

$$\begin{aligned}\text{var}(Y_f|X=0) = & (\gamma_b+\gamma)^2\text{var}(U|X=0) + (\delta_b+\delta)^2\text{var}(C|X=0) + \\ & \text{var}(\varepsilon_b|X=0) + \text{var}(\varepsilon_f|X=0) + 2\text{cov}(\varepsilon_b,\varepsilon_f|X=0)+\\ & 2(\gamma+\gamma_b)(\delta+\delta_b)\text{cov}(U,C|X=0)\end{aligned} \tag{B.3}$$

Similarly, the variance of $Y_f$ in a the intervention arm ($X = 1$) is given by

$$\begin{aligned}\text{var}(Y_f|X=0) =&\gamma_b^2\text{var}(U|X=1)+\delta_b^2\text{var}(C|X=1)+\text{var}(\varepsilon_b|X=1)+\\&2\gamma_b\delta_b\text{cov}(U,C|X=1)+2\gamma_b\text{cov}(U,\varepsilon_Y|X=1)+2\delta_b\text{cov}(C,\varepsilon_Y|X=1)+\\&2\gamma_b\gamma\text{cov}(U,U|X=1)+2\gamma_b\delta\text{cov}(U,C|X=1)+2\gamma_b\zeta\text{cov}(U,S|X=1)+\\&2\gamma_b\text{cov}(U,\varepsilon_Y|X=1)+2\delta_b\zeta\text{cov}(C,S|X=1)+2\delta_b\delta\text{cov}(C,C|X=1)+\\&2\delta_b\text{cov}(C,\varepsilon_Y|X=1)+2\zeta\text{cov}(S\varepsilon_f,|X=1)+2\text{cov}(\varepsilon_b,\varepsilon_f|X=1)+\\&\gamma^2\text{var}(U|X=1)+\delta^2\text{var}(C|X=1)+\zeta^2\text{var}(S|X=1)+\text{var}(\varepsilon_f|X=1)+\\&2\gamma\delta\text{cov}(U,C|X=1)+2\gamma\zeta\text{cov}(U,S|X=1)+2\gamma\text{cov}(U,\varepsilon_Y|X=1)+\\&2\delta\zeta\text{cov}(C,S|X=1)+2\delta\text{cov}(C,\varepsilon_Y|X=1)+2\zeta\text{cov}(S,\varepsilon_Y|X=1).\end{aligned} \tag{B.4}$$

With $C$, $U$, $S$ all independent of $\varepsilon_f$, and $S$ independent of $U$ and $C$, (B.4) reduces to equation (20) of the main text):

$$\begin{aligned}\text{var}(Y_f|X=1)=&(\gamma_b+\gamma)^2\text{var}(U|X=1)+(\delta_b+\delta)^2\text{var}(C|X=1)+\\&\text{var}(\varepsilon_b|X=1)+\zeta^2\text{var}(S|X=1)+\text{var}(\varepsilon_f|X=1)+\\&2\text{cov}(\varepsilon_b,\varepsilon_f|X=1)+(\gamma+\gamma_b)(\delta+\delta_b)\text{cov}(U,C|X=1).\end{aligned} \tag{B.5}$$

With $\varepsilon_b$ and $\varepsilon_f$ independent of $X$, the outcome variance difference across trial arms in the full data is then given by the difference of (B.3) and (B.5) (equation (21) of the main text):

$$\text{VD}_f=\text{var}(Y_f|X=1)-\text{var}(Y_f|X=0)=\zeta^2\text{var}(S|X=1). \tag{B.6}$$

With $S \perp\!\!\!\perp C$, the covariate adjusted variance difference, $\text{VD}_{f(\text{C})}$=$\text{VD}_f$. The outcome variance difference is non zero, due to the presence of effect modifier, $S$, which violates assumption A1. In the absence of effect modification, $\zeta=0$, and $\text{VD}_f$=0.

Note that if $S$ is related to $C$ and $U$, then (B.5) will instead be given by

$$\begin{aligned}\text{var}(Y_f|X=1)=&(\gamma_b+\gamma)^2\text{var}(U|X=1)+(\delta_b+\delta)^2\text{var}(C|X=1)+\\&\text{var}(\varepsilon_b|X=1)+\zeta^2\text{var}(S|X=1)+\text{var}(\varepsilon_f|X=1)+\\&2\text{cov}(\varepsilon_b,\varepsilon_f|X=1)+(\gamma+\gamma_b)(\delta+\delta_b)\text{cov}(U,C|X=1)+\\&2\zeta(\gamma+\gamma_b)\text{cov}(S,U|X=1)+2\zeta(\delta+\delta_b)\text{cov}(S,C|X=1),\end{aligned} \tag{B.7}$$

and (B.6) by

$$\text{VD}_f=\zeta^2\text{var}(S|X=1)+2\zeta(\gamma+\gamma_b)\text{cov}(S,U|X=1)+2\zeta(\delta+\delta_b)\text{cov}(S,C|X=1), \tag{B.8}$$

with additional covariance terms accounting for the dependency between $S$ and $U$ and between $S$ and $C$.

### B.2 Heteroscedastic errors (assumption 2 is violated)

Here, we show that when assumption A2 is violated, resulting in heteroscedastic outcome errors, the outcome variances across trial arms in the full data are different at follow-up but not at baseline. Let the outcome at baseline, $Y_b$, be given by:

$$Y_b = \alpha_b + \gamma_b U + \delta_b C + \varepsilon_b, \tag{B.9}$$

with $U$, $C$, and the coefficients defined as in equation (14) of the main text, but now with $\varepsilon_b$ no longer independent of $Y_b$.

Let the outcome at follow-up, $Y_f$ by given by

$$Y_f = Y_b + \beta X + \gamma U + \delta C + \varepsilon_f, \tag{B.10}$$

with $X$, $U$, $C$, and the coefficients defined as in equation (17) of the main text, but now with $\varepsilon_f$ no longer independent of $Y_f$. Note that equation (17) of the main text contains an additional term '$\zeta SX$'; here we have set the coefficient, $\zeta$, to 0 so that there is no treatment effect heterogeneity. As in Section 5.1 of the main text, $Y_b$ and $Y_f$ are part of a time series and $\varepsilon_b$ and $\varepsilon_f$ are correlated.

$Y_b$ is unaffected by $X$, and, consequently, the outcome variance difference across trial arms at baseline, $\text{VD}_b = 0$ (equation (16) of the main text). For $Y_f$, the outcome variances in both trial arms can be written as in (B.3), with, for a given arm, $j$:

$$\begin{aligned}\text{var}(Y_f|X=j) = &(\gamma_b+\gamma)^2\text{var}(U|X=j) + (\delta_b+\delta)^2\text{var}(C|X=j) + \\ &\text{var}(\varepsilon_b|X=j) + \text{var}(\varepsilon_f|X=j) + 2\text{cov}(\varepsilon_b,\varepsilon_f|X=j) + \\ &(\gamma+\gamma_b)(\delta+\delta_b)\text{cov}(U,C|X=j)\end{aligned} \tag{B.11}$$

Given that a treatment effect is present so that $\beta \neq 0$ in (B.10), and with $\varepsilon_f$ dependent on $Y_f$, which is affected by $X$, then, in (B.11), $\text{var}(\varepsilon_f|X=1) \neq \text{var}(\varepsilon_f|X=0)$ and $\text{cov}(\varepsilon_b,\varepsilon_f|X=1) \neq \text{cov}(\varepsilon_b,\varepsilon_f|X=0)$. Consequently, the outcome variance difference at follow-up, $\text{VD}_f$, is non-zero, and given by

$$\begin{aligned}\text{VD}_f = &\text{var}(Y_f|X=1) - \text{var}(Y_f|X=0) = \\ &\text{var}(\varepsilon_f|X=1) - \text{var}(\varepsilon_f|X=0) + \\ &2\big(\text{cov}(\varepsilon_b,\varepsilon_f|X=1) - \text{cov}(\varepsilon_b,\varepsilon_f|X=0)\big).\end{aligned}$$

# C Simulation study of Table 2, Section 5

Here, we describe the simulation approach employed to generate the data used to obtain the results in Table 2 of the main text. This table shows the bias of the treatment effect, as estimated by a CCA linear regression and the outcome variance difference across trial arms at follow-up and at baseline, as estimated by the studentized Breusch-Pagan test, across seven dropout scenarios, shown as DAGs in Figure 1 of the main text. Section C.1 describes the simulation approach in detail, using the ADEMP framework [1]. In Section C.2, a companion table is provided for Table 2 of the main text.

## C.1 Simulation approach

Here, we describe the simulation approach in detail using the ADEMP framework [1], and define the aims, data-generating mechanisms, estimands, methods, and performance measures.

**Aim**: Illustrating that, for longitudinal data, 1) a variance difference in outcomes at follow-up may be the result of outcome-dependent dropout, non-outcome dependent dropout or treatment effect heterogeneity (resulting from effect modification); 2) a variance difference in outcomes at baseline only results from outcome-dependent dropout.

**General setup:** Longitudinal data with correlated outcomes at baseline and follow-up, drawn from a multivariate normal distribution, simulated at a sample size of $N = 1000$, with balanced and unbalanced randomization to intervention and comparator arm and a positive treatment effect, for seven different dropout scenarios (see Table 1 and Figure 1 of the main text), simulated using a logit mechanism.

Data generating mechanism:

**Variables:** Binary treatment variable, $X \sim B(1000, 0.50)$, with half of 2000 patients randomized to the intervention arm (X=1) and half to the comparator arm (X=0); a normally distributed variable $C \sim N(0, \sqrt{0.5})$ and a normally distributed variable $U \sim N(0, \sqrt{2})$, which affect the outcome at final follow-up, $Y_f$ (equation (17) of the main text), and the outcome at baseline, $Y_b$ (equation (14) of the main text); normally distributed variable $S \sim N(1, \sqrt{2})$, which modifies the effect of $X$ on $Y_f$. $Y_f$ and $Y_b$ are drawn from a multivariate normal distribution. $Y_b$ is given by

$$Y_b = 0.25 + 0.5C + 0.5U + \varepsilon_b,$$

and $Y_f$ by

$$Y_f = 0.25 + Y_b + \beta X + C + U + \zeta SX + \varepsilon_f.$$

In the simulation, we consider two cases of $Y_f$:

1. $\zeta = 0$ so that there is no effect modification, and the treatment effect is equal to $\beta$, with $\beta = 1$

2. $\zeta = 1$ so that $S$ modifies the effect of $X$ on $Y_f$ in the intervention arm. We specify $\beta = 0.5$ for the unmodified effect of $X$ on $Y_f$. Then, the average treatment effect, $\beta_{av}$, which we estimate in a linear regression of $Y_f$ on $X$, $C$, and, optionally, $Y_b$ (see 'Estimands'), is given by $\beta_{av} = \beta + \zeta = 0.5 + 0.5 = 1$

The $Y_b$ error, $\varepsilon_b$, and the $Y_f$ error, $\varepsilon_f$, are drawn from a multivariate normal distribution:

$$\begin{pmatrix} \varepsilon_b \\ \varepsilon_f \end{pmatrix} \sim N\left( \begin{pmatrix} 0 \\ 0 \end{pmatrix}, \begin{pmatrix} 1.5 & 0.75 \\ 0.75 & 2 \end{pmatrix} \right),$$

The outcome variance in a given trial arm, $j$, at baseline, is then $\text{var}(Y_b|X = j) = \text{var}(0.5U + 0.5C + \varepsilon_b|X = j) = 2.125$. The outcome variance at follow-up is affected by the absence/presence of effect modification:

1. In the absence of effect modification ($\zeta = 0$), the outcome variance in a given trial arm, $j$, at follow-up, is then: $\text{var}(Y_f|X = j) = \text{var}(Y_b + U + C + \varepsilon_f|X = j) = 10.625$
2. In the presence of effect modification ($\zeta = 0.5$), the outcome variance at follow-up in the comparator arm is shown above, and in the intervention arm is given by: $\text{var}(Y_f|X = j) = \text{var}(Y_b + U + C + 0.5S + \varepsilon_f|X = j) = 11.625$

Results are reported in the main text, Table 2 and in Supplementary Table C.1).

**Dropout mechanism:** Logit selection mechanism with intercepts selected such that overall dropout proportion is approximately 0.27 to 0.29, with seven different dropout scenarios considered (see Table 1 of the main text, also shown as directed acyclic graphs (DAGs) A to G in Figure 1 of the main text). For each scenario, the probability of selection is defined conditional on the model variables: $\mathsf{P}(R = 1|Y_f, X, C, U)$, with $R$ the response indicator, with 1 indicating the outcome at follow-up is observed for and 0 that it is not.

A *Random dropout*

- $\mathsf{P}(R = 1|Y_f, X, U) = 0.73$

B *X-dependent dropout*

- $\mathsf{P}(R = 1|Y_f, X, CU) = \exp(0.2 + 2X)/(1 + \exp(0.2 + 2X)$

C *C-dependent dropout*

- $\mathsf{P}(R = 1|Y_f, X, CU) = \exp(-0.7 + 2C)/(1 + \exp(-0.7 + 2C)$

D *$C$- and $X$-dependent dropout*

- $\mathsf{P}(R=1|Y_f,X,C,U) = \exp(-1.55+2C+2X)/(1+\exp(-1.55+2C+2X)$

E *$Y_f$-dependent dropout*

- $\mathsf{P}(R=1|Y_f,X,C,U) = \exp(-3.8+2Y_f)/(1+\exp(-3.8+2Y_f)$

F *$U$-dependent dropout*

- $\mathsf{P}(R=1|Y_f,X,CU) = \exp(2U)/(1+\exp(2U)$

G *Non-$Y_f$-dependent MNAR dropout ($X$- and $U$-dependent*

- $\mathsf{P}(R=1|Y_f,X,C,U) = \exp(-0.9+2X+2U)/(1+\exp(-0.9+2X+2U)$

**Simulation size:** For each scenario, 1000 datasets were simulated. Simulation quality was verified by checking of mean SEs of the CCA and variance difference estimates were comparable to the Monte Carlo SDs (see 'Performance measures')

**Estimands**
*Primary:* Reported in main text, Table 2.

- Bias of the complete case analysis (CCA) treatment effect estimate, regressing outcome, $Y_f$, on treatment, $X$, and covariate, $C$, with 95% CI
- Bias of the adjusted CCA treatment effect estimate, regressing outcome, $Y_f$, on treatment, $X$, covariate, $C$, and the outcome at baseline, $Y_b$, with 95% CI
- Outcome variance difference across trial arms at follow-up in the observed data, adjusted for $C$, with 95% CI, and proportion of times the null hypothesis of no variance difference was rejected
- Outcome variance difference across trial arms at follow-up in the observed data, adjusted for $C$ and outcome at baseline, $Y_b$, with 95% CI,and proportion of times the null hypothesis of no variance difference was rejected
- Unadjusted outcome variance difference across trial arms at baseline in the observed data, with 95% CI, and proportion of times the null hypothesis of no variance difference was rejected

*Secondary:* Reported in appendix, Table C.1.

- All previous items
- Standard error (SE) of all five measures listed above
- Monte Carlo SD (MCSD) of all five measures listed above

- Dropout proportions across trial arms and overall dropout proportion

**Methods**

- CCA estimator
- Studentized Breusch-Pagan test (for estimating the variance difference across groups after dropout)
- 95% CIs were calculated using the Monte Carlo Standard deviation (MCSD)

**Performance measures**: Simulation quality was checked by seeing if the estimate SEs were comparable to the MCSDs

## C.2 Simulation results

Table C.1 is a companion table to Table 2 in the main text, and additionally reports, for each estimate, the standard error (SE), the Monte Carlo SD (MCSD), and the dropout proportion in the comparator arm ($d_0$), the trial arm ($d_1$) and the overall dropout proportion ($d_t$). We observe that the SEs and MCSDs are comparable, suggesting that a simulation size of 1000 is sufficient.

**Table C.1:** Companion table to Table 2 of the main text. Bias of the complete case analysis (CCA) treatment effect estimate, obtained in a linear regression of $Y_f$ on $X$, $C$, and, optionally, $Y_b$; variance difference (VDs) across trial arms in the outcome at baseline, in the observed data ($\text{VD}^*_b$); variance difference across trial arms in the outcome at follow-up, estimated conditional on $C$ ($\text{VD}^*_{f(\text{C})}$), and conditional on $C$ and $Y_b$ ($\text{VD}^*_{f(\text{C},Yb)}$). Additionally shown, for each estimate, are the 95% confidence interval (CI), standard error (SE), Monte Carlo SD (MCSD), proportion of 95% CIs excluding the null (e.g., $p_b$, for $\text{VD}_b$), dropout proportions in the intervention ($d_1$) and comparator ($d_0$) groups and the overall dropout proportion ($d_t$). The simulated data is longitudinal, with baseline ($Y_b$) and follow-up ($Y_f$) measurements, simulated under seven different dropout mechanisms (see Table 1 and Figure 1 of the main text), with and without effect modification (EM). The estimates shown are mean values across 1000 simulated datasets of $N = 2000$, with, in the absence of effect modification, a true treatment effect $\beta = 1$, and trial arm outcome variances at baseline and follow-up of 2.125 and 10.625, respectively. In the presence of effect modification, $\beta_{av} = 1$, and the outcome variance at follow-up in the intervention arm is 10.875.

| | Bias (95% CI) | SE | MCSD | $\text{VD}^*_b$ (95% CI) | SE | MCSD | $p_b$ | $\text{VD}^*_{f(\text{C})}$ (95% CI) | SE | MCSD | $p_{f(\text{C})}$ |
|---|---|---|---|---|---|---|---|---|---|---|---|
| A | 0.00 (-0.33,0.33) | 0.16 | 0.17 | 0.00 (-0.30,0.30) | 0.16 | 0.15 | 0.04 | -0.01 (-1.37,1.35) | 0.70 | 0.69 | 0.04 |
| A(EM) | 0.00 (-0.33,0.34) | 0.17 | 0.17 | 0.00 (-0.30,0.30) | 0.16 | 0.15 | 0.04 | 1.00 (-0.44,2.44) | 0.74 | 0.73 | 0.26 |
| B | 0.00 (-0.34,0.34) | 0.17 | 0.17 | 0.00 (-0.31,0.30) | 0.16 | 0.16 | 0.04 | 0.01 (-1.38,1.39) | 0.73 | 0.71 | 0.03 |
| B(EM) | 0.00 (-0.34,0.34) | 0.17 | 0.17 | 0.00 (-0.31,0.30) | 0.16 | 0.16 | 0.04 | 1.01 (-0.42,2.43) | 0.78 | 0.73 | 0.25 |
| C | 0.00 (-0.32,0.32) | 0.16 | 0.16 | 0.00 (-0.31,0.30) | 0.16 | 0.15 | 0.04 | -0.01 (-1.38,1.35) | 0.71 | 0.70 | 0.05 |
| C(EM) | 0.00 (-0.32,0.32) | 0.17 | 0.16 | 0.00 (-0.31,0.30) | 0.16 | 0.15 | 0.04 | 0.99 (-0.42,2.40) | 0.75 | 0.72 | 0.27 |
| D | 0.00 (-0.33,0.33) | 0.17 | 0.17 | 0.01 (-0.30,0.32) | 0.16 | 0.16 | 0.05 | 0.02 (-1.38,1.43) | 0.72 | 0.72 | 0.04 |
| D(EM) | 0.00 (-0.33,0.33) | 0.17 | 0.17 | 0.01 (-0.30,0.32) | 0.16 | 0.16 | 0.05 | 1.03 (-0.43,2.49) | 0.77 | 0.75 | 0.26 |
| E | -0.42 (-0.66,-0.17) | 0.12 | 0.12 | 0.12 (-0.09,0.33) | 0.11 | 0.11 | 0.20 | 0.71 (-0.12,1.55) | 0.43 | 0.43 | 0.36 |
| E(EM) | -0.31 (-0.56,-0.07) | 0.13 | 0.13 | 0.16 (-0.05,0.38) | 0.11 | 0.11 | 0.32 | 1.20 (0.32,2.07) | 0.45 | 0.45 | 0.76 |
| F | 0.00 (-0.30,0.29) | 0.15 | 0.15 | 0.00 (-0.29,0.29) | 0.14 | 0.15 | 0.06 | 0.01 (-1.15,1.17) | 0.59 | 0.59 | 0.05 |
| F(EM) | -0.01 (-0.31,0.30) | 0.15 | 0.15 | 0.00 (-0.29,0.29) | 0.14 | 0.15 | 0.06 | 1.02 (-0.21,2.24) | 0.63 | 0.63 | 0.37 |
| G | -0.52 (-0.82,-0.22) | 0.15 | 0.15 | 0.06 (-0.22,0.34) | 0.15 | 0.14 | 0.06 | 0.56 (-0.61,1.73) | 0.60 | 0.60 | 0.16 |
| G(EM) | -0.52 (-0.82,-0.22) | 0.16 | 0.15 | 0.06 (-0.22,0.34) | 0.15 | 0.14 | 0.06 | 1.56 (0.32,2.79) | 0.65 | 0.63 | 0.69 |

| | Bias$\|Y_b$ (95% CI) | SE | MCSD | $\text{VD}^*_{f(\text{C},Yb)}$ | SE | MCSD | $p_{f(\text{C},Yb)}$ | $d_0$ | $d_1$ | $d_t$ |
|---|---|---|---|---|---|---|---|---|---|---|
| A | 0.00 (-0.16,0.17) | 0.08 | 0.08 | 0.00 (-0.36,0.36) | 0.18 | 0.18 | 0.05 | 0.27 | 0.27 | 0.27 |
| A(EM) | 0.00 (-0.18,0.18) | 0.09 | 0.09 | 1.00 (0.57,1.44) | 0.22 | 0.22 | 1.00 | 0.27 | 0.27 | 0.27 |
| B | 0.00 (-0.17,0.17) | 0.09 | 0.08 | 0.01 (-0.37,0.38) | 0.19 | 0.19 | 0.06 | 0.45 | 0.10 | 0.27 |
| B(EM) | 0.00 (-0.18,0.17) | 0.10 | 0.09 | 1.01 (0.58,1.43) | 0.24 | 0.22 | 1.00 | 0.45 | 0.10 | 0.27 |
| C | 0.00 (-0.15,0.16) | 0.08 | 0.08 | 0.00 (-0.35,0.36) | 0.18 | 0.18 | 0.05 | 0.28 | 0.28 | 0.28 |
| C(EM) | 0.00 (-0.17,0.17) | 0.09 | 0.09 | 1.00 (0.58,1.43) | 0.22 | 0.22 | 1.00 | 0.28 | 0.28 | 0.28 |
| D | 0.00 (-0.16,0.16) | 0.09 | 0.08 | 0.00 (-0.36,0.37) | 0.19 | 0.19 | 0.05 | 0.42 | 0.14 | 0.28 |
| D(EM) | 0.00 (-0.18,0.18) | 0.10 | 0.09 | 1.00 (0.58,1.43) | 0.24 | 0.22 | 0.99 | 0.42 | 0.14 | 0.28 |
| E | -0.15 (-0.31,0.00) | 0.08 | 0.08 | 0.11 (-0.19,0.4) | 0.15 | 0.15 | 0.10 | 0.32 | 0.22 | 0.27 |
| E(EM) | -0.05 (-0.22,0.11) | 0.08 | 0.08 | 0.83 (0.47,1.19) | 0.19 | 0.18 | 1.00 | 0.32 | 0.23 | 0.27 |
| F | 0.00 (-0.15,0.16) | 0.08 | 0.08 | 0.00 (-0.32,0.32) | 0.16 | 0.16 | 0.05 | 0.27 | 0.27 | 0.27 |
| F(EM) | 0.00 (-0.17,0.17) | 0.09 | 0.09 | 1.00 (0.61,1.39) | 0.21 | 0.20 | 1.00 | 0.27 | 0.27 | 0.27 |
| G | -0.21 (-0.36,-0.05) | 0.08 | 0.08 | 0.09 (-0.23,0.42) | 0.17 | 0.17 | 0.09 | 0.37 | 0.18 | 0.27 |
| G(EM) | -0.21 (-0.38,-0.04) | 0.09 | 0.09 | 1.09 (0.70,1.49) | 0.21 | 0.2 | 1.00 | 0.37 | 0.18 | 0.27 |

# D Simulation study of Table 3, Section 6

Here, we describe the simulation approach employed to generate the data used in Table 3 of Section 6 of the main text. This table shows the bias of the treatment effect, as estimated by a CCA linear regression and by a multiple imputation (MI) model, and the outcome variance difference across trial arms, as estimated by the studentized Breusch-Pagan test, across two dropout scenarios, shown as DAGs in Figure 1 of the main text. Section D.1 describes the simulation approach in detail, using the ADEMP framework[1]. Section D.2 gives the results for a simulation at sample sizes of $N = 1000$ and $N = 10000$, with Table D.1 a companion table to Table 3 of the main text.

## D.1 Simulation approach

Here, we describe the simulation approach in detail using the ADEMP framework[1], and define the <u>a</u>ims, <u>d</u>ata-generating mechanisms, <u>e</u>stimands, <u>m</u>ethods, and <u>p</u>erformance measures.

**Aim**: Illustrating that 1) when performing a multiple imputation (MI) analysis, the variance difference may be used to assess the added value of including variables in the imputation model; 2) if dropout is MAR conditional on the imputation model covariates, then the treatment effect, estimated with the analysis model is unbiased and the variance difference, conditional on the analysis model (close to) zero; 3) if dropout is MNAR conditional on the imputation model covariates, then the treatment effect, estimated with the analysis model is biased and the variance difference, conditional on the analysis model non-zero.

**General setup:** Data with normally distributed outcomes, simulated with a treatment effect of $\beta = 1$, at two different sample sizes, for two different dropout scenarios, simulated with a logit mechanism.

<u>Data generating mechanism:</u>

**Variables**: Binary treatment variable, $X \sim B(1, 0.50)$, some normally distributed variables $C_1 \sim N(0, \sqrt{2})$ and $C_2 \sim N(0, \sqrt{2})$, which affect $Y$, the outcome at final follow-up so that $Y = \beta X + C_1 + C_2 + \varepsilon$, with a positive treatment effect of $\beta = 1$, and $\varepsilon \sim N(0, \sqrt{4})$ so that the outcome variance per trial arm in the full data is $\text{var}(Y|X = j) = \text{var}(C_1 + C_2 + \varepsilon|X = j) = 8$. (Results reported in the main text, Table 3, and in Appendix D.2, Table D.1)

**Sample size**: Total sample sizes of $N = 1000$ and $N = 10000$

**Dropout mechanism:** Logit selection mechanism with intercepts selected such that overall dropout proportion is approximately 0.27, with two different dropout scenarios considered (shown as directed acyclic graphs (DAGs) 1 and 2 in Figure 2 of the main text). For each scenario, the probability of selection is defined conditional on the model variables: $\mathsf{P}(R = 1|Y, X, C_1, C_2)$, with $R$ the response indicator, with 1 indicating the outcome at follow-up is observed and 0 that it is not.

M1 *MAR dropout dependent on $C_2$ and $X$*

- $\mathsf{P}(R = 1|Y, X, C_1, C_2) = \exp(1.1 + 2.1X)/(1 + \exp(1.1 + 2.2X)$

M2 *MNAR dropout dependent on $C_2$, $X$, and $Y$*

- $\mathsf{P}(R = 1|Y, X, C_1, C_2) = \exp(2.8+2.1X+2.1B+2.1Y)/(1+\exp(2.8+2.1X+2.1B + 2.1Y))$

**Simulation size:** For each scenario, 1000 datasets were simulated. Simulation quality was verified by checking of mean SEs of the CCA and variance difference estimates were comparable to the Monte Carlo SDs (see 'Performance measures')

**Estimands**
*Primary:* Reported in main text, Table 3, and in appendix, Table D.1.

- Bias of the complete case analysis (CCA) treatment effect estimate, regressing outcome, $Y$, on treatment, $X$, and covariate $C_1$, with 95% CI.
- Outcome variance difference across trial arms in the observed datam adjusted for $C_1$ , with 95% CI.
- Bias of the MI treatment effect estimate, regressing outcome, $Y$, on treatment, $X$, and covariate $C_1$, in datasets with outcomes imputed with an imputation model including $Y$, $X$, $C_1$ and $C_2$, with 95% CI.
- Outcome variance difference across trial arms in the observed data, adjusted for $C_1$, estimated in datasets with outcomes imputed with an imputation model including $Y$, $X$, $C_1$ and $C_2$, with 95% CI.

*Secondary:* Reported in appendix, Table D.1.

- Proportion of times the null hypothesis of no bias or no variance difference was rejected, calculated by counting the times the null value was excluded from the 95% CI.
- Standard error (SE) of all four measures listed above
- Monte Carlo SD (MCSD) of all four measures listed above

**Methods**

- CCA estimator
- 10-fold multiple imputation, performed using *R* software package 'mice'
- Studentized Breusch-Pagan test (for estimating the variance difference across groups after dropout in the observed data, and in the imputed datasets)
- 95% CIs were calculated using the Monte Carlo Standard deviation (MCSD)

**Performance measures**: Simulation quality was checked by seeing if the mean estimate SEs were comparable to the MCSDs

## D.2 Simulation results

Table D.1 is a companion table to Table 3 of the main text, and reports the results for a samples size of $N = 1000$ and $N = 10000$. Additionally, the table reports, for each estimate, the standard error (SE), the Monte Carlo SD (MCSD), and the dropout proportion in the comparator arm ($d_0$), the trial arm ($d_1$) and the overall dropout proportion ($d_t$). We observe that the SEs and MCSDs are comparable, suggesting that a simulation size of 1000 is sufficient.

**Table D.1:** Companion table to Table 3 of the main text. Bias of the complete case analysis (CCA) and multiple imputation (MI) treatment effect estimates and outcome variance differences (VDs) across trial arms in the observed data, for data ($N = 1000$ and $N = 10000$) simulated according to directed acyclic graphs (DAGs) 1 and 2 (Figure 2)). 1) $Y$ is a function of $C_1$, $C_2$, and $X$, with dropout dependent on $C2$ and $X$; 2) Analogous to 1, with dropout additionally dependent on $Y$. Shown is the CCA treatment effect estimate, conditional on $C_1$, with corresponding VD in the observed data (VD$^*$), alongside the MI treatment effect estimate and VD (VD$^*_{\text{MI}}$), estimated conditional on $C_1$, with both $C_1$ and $C_2$ included in the imputation model. Shown for each are the 95% CI, standard error (SE), and Monte Carlo standard deviation (MCSD). Additionally shown are the dropout proportions in the comparator arm ($d_0$), intervention arm ($d_1$), the overall dropout proportion ($d_t$), and, for the VD in the observed data and the VD in the imputed data, the proportion of times the null hypothesis of no variance difference was rejected ($p_{\text{CCA}}$ and $p_{\text{MI}}$, respectively). The estimates shown are mean values across 1000 simulated datasets, with a true treatment effect $\beta = 1$, and trial arm outcome variances of 8.

| | *Observed data* | | | | | | | *Dropout* | | |
|---|---|---|---|---|---|---|---|---|---|---|
| | Bias (95% CI) | SE | MCSD | VD$^*$ (95% CI) | SE | MCSD | $p_{\text{CCA}}$ | $d_0$ | $d_1$ | $d_t$ |
| *N=1000* | | | | | | | | | | |
| 1 | -0.36 (-0.70,-0.01) | 0.17 | 0.18 | 0.28 (-0.84,1.40) | 0.56 | 0.57 | 0.08 | 0.38 | 0.18 | 0.28 |
| 2 | -0.56 (-0.84,-0.27) | 0.15 | 0.15 | 0.72 (-0.08,1.53) | 0.41 | 0.41 | 0.42 | 0.36 | 0.19 | 0.28 |
| *N=10000* | | | | | | | | | | |
| 1 | -0.35 (-0.46,-0.25) | 0.05 | 0.05 | 0.26 (-0.09,0.61) | 0.18 | 0.18 | 0.31 | 0.37 | 0.18 | 0.28 |
| 2 | -0.56 (-0.65,-0.47) | 0.05 | 0.05 | 0.72 (0.46,0.98) | 0.13 | 0.13 | 1.00 | 0.36 | 0.19 | 0.28 |
| | *Imputed data* | | | | | | | | | |
| | Bias (95% CI) | SE | MCSD | VD$^*_{\text{MI}}$ (95% CI) | SE | MCSD | $p_{\text{MI}}$ | | | |
| *N=1000* | | | | | | | | | | |
| 1 | -0.02 (-0.38,0.34) | 0.18 | 0.18 | 0.08 (-0.85,1.01) | 0.61 | 0.48 | 0.02 | | | |
| 2 | -0.48 (-0.75,-0.22) | 0.13 | 0.13 | 0.75 (0.11,1.38) | 0.36 | 0.32 | 0.52 | | | |
| *N=10000* | | | | | | | | | | |
| 1th | 0.00 (-0.11,0.11) | 0.06 | 0.06 | 0.02 (-0.28,0.32) | 0.20 | 0.15 | 0.01 | | | |
| 2th | -0.48 (-0.56,-0.4) | 0.04 | 0.04 | 0.75 (0.54,0.95) | 0.12 | 0.1 | 1.00 | | | |

# E Supplementary Table E.1 (Section 7.1)

**Table E.1:** Standard deviations (SDs) of the headache score at baseline, across six bins. The number of patients per bin is denoted 'N'. The data used is from an RCT on the benefit of acupuncture treatment versus usual care in 401 patients suffering from chronic headaches.

| | (6.66,21.4] | (21.4,36.1] | (36.1,50.8] | (50.8,65.4] | (65.4,80.1] | (80.1,94.8] |
|---|---|---|---|---|---|---|
| N | 206 | 104 | 51 | 26 | 12 | 2 |
| SD | 3.78 | 4.21 | 4.36 | 4.46 | 4.14 | 5.13 |

# References

[1] Morris TP, White IR, Crowther MJ. Using simulation studies to evaluate statistical methods. *Statistics in Medicine* 2019; 38(11): 2074-2102. doi: https://doi.org/10.1002/sim.8086
